# Harnessing polysaccharide-mediated biomineralization for advanced bone tissue engineering

**DOI:** 10.1016/j.mtbio.2026.102846

**Published:** 2026-01-23

**Authors:** Huxin Tang, Mingyang Hu, Xinying Huang, Jianan Chen, Yesheng Jin, Shuo Chen, Ke Li, Yong Xu

**Affiliations:** a Department of Orthopaedics, The First Affiliated Hospital of Soochow University, MOE Key Laboratory of Geriatric Diseases and Immunology, Suzhou Medical College, Soochow University, Suzhou, Jiangsu, 215000, China; b Department of Sports Medicine, Nanjing Hospital of Chinese Medicine affiliated to Nanjing University of Chinese Medicine, Nanjing, 210022, China; c Department of Burn and Plastic Surgery, The First Affiliated Hospital of Soochow University, Soochow University, Suzhou, 215006, China; d National Center for Translational Medicine (Shanghai) SHU Branch, Shanghai University, Shanghai, 200444, China

**Keywords:** Biomineralization, Polysaccharide, Bone tissue engineering, Hydroxyapatite, Regulation of mineralization

## Abstract

Biomineralization is a critical process wherein organisms form mineral composites via organic-inorganic synergistic interactions, which are essential for maintaining and repairing bone tissue homeostasis. Polysaccharides, as a class of natural biological macromolecules, play a crucial role in regulating biomineralization processes. This may be ascribed to their distinctive physical and chemical characteristics, in addition to their biological functions. These molecules effectively alter the crystalline structure and mechanical attributes of minerals like hydroxyapatite by adjusting ion levels, supplying sites for nucleation during mineral formation, and interacting with other biomolecules such as collagen to direct the deposition of minerals. Chitosan, alginate, hyaluronic acid, and sulfated polysaccharides have shown significant biomimetic properties through the creation of biomimetic scaffolds, improvement of cell attachment and differentiation, and facilitation of bone defect healing. This article systematically reviews the molecular mechanisms of polysaccharides in biomineralization and discusses their applications in bone tissue engineering from a biomineralization perspective, thereby offering novel insights for clinical treatment.

## Graphical abstract

**Figure:**
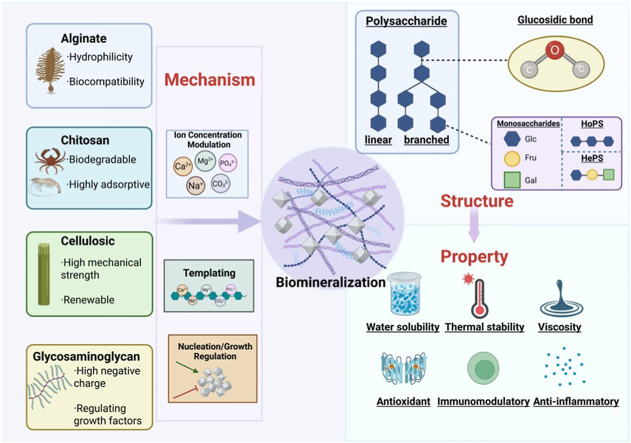

## 1 Introduction

The intensifying global aging trends have made bone mineralization disorders, such as osteoporosis and bone defects of different degrees, more and more prominent public health challenges. Epidemiological research data show that approximately 20 % of adults in Europe suffer from these conditions [1]. The occurrence rate of critical-sized bone defects has also risen notably. This is closely related to factors like high-energy trauma, chronic diseases, and changes in lifestyle [2]. These diseases directly undermine the mineral deposition capacity of bone tissue, leading to mechanical malfunction and regeneration difficulties. Therefore, there is an urgent need for effective treatment approaches.

At present time, there are some classic approaches in the clinic treatment of bone defect and they all have their inherent shortcomings: Autogenous bone transplantation is regarded as "golden standard", which requires extracting of the bone tissue from a human's body such as an iliac bone [3]. However, despite having a good osteogenic ability as well as biocompatibility, its drawback lies in the limited availability of donors, complications at the donor site, risks for chronic pain and prolonged surgery time. Allogeneic bone grafts come from bone banks, which solves the issue of donors' shortage; but raise questions on immunity reactions and possible contagion transmissions, and slow incorporation because of the lower biological activity [4]. Metallic implants provide instantaneous mechanical support to load-bearing defects; however, they are biostatic and nonbiodegradable nature may lead to mechanical mismatch, which can lead to stress shielding, peri-prosthetic bone resorption, and often requires a secondary resection surgery.

Bone tissue engineering is considered to be an ideal substitute treatment approach in order to circumvent some intrinsic disadvantages of conventional therapeutic approaches. The development of biofunctional scaffolds able to provide mechanical stability and stimulate bone regeneration, this discipline seeks to go beyond simple tissue substitution, aiming for a true physiological replacement.

While there has been major progress towards the provision of structural support to damaged bone by the range of tissue engineered materials that are now available, however, it is still limited to simulate functional regeneration like real bone tissues. For example, synthetic polymers like poly lactic-co-glycolic acid (PLGA) or polycaprolactone (PCL), as well as bio-inert metal implants, mainly act as inert filler materials, without any inherent capacity for active promotion of bone-specific mineralization. In vitro experiments, their hydroxyapatite's depositing efficiency and biomimetic degree are poor; directly impairing the effectiveness of osseointegration of implants [5]. With regard to vascularization, as bone tissue needs rich vascularization in order to survive, the failure of pre-vascularization becomes one of the key bottlenecks for repairing bone defects. Over 30 % of failed bone defect repair is due to bone resorption as a result of insufficient pre-vascularization, especially for large segmental defects [6]. They can't sense and respond to important regenerative microenvironmental signals, such as hypoxia-inducible factor (HIF-1a) and vascular endothelial growth factor (VEGF), nor are they capable to dynamically orchestrate a number of repairing procedures such as osteogenesis, angiogenesis etc., which essentially limits the efficiency and quality of functional bone regeneration [7].

Here, polysaccharide-based approaches constitute a smart multifunctional system capable of directing and modulating new bone formation. Unlike passive scaffolds, this progress is first manifested in the biomorphic and active character of the mineralization process: unlike passive implants, polysaccharides like chitosan with the help of their functionalities (-NH_2_, -OH etc.) can act as a dynamic trap for ions and also serve as template molecules, not only efficiently concentrating calcium and phosphate ions by means of electrostatic attraction but also (via special spatial conformations) directing the nucleation, stabilization, and ordered transformation of amorphous mineral precursors (e.g., amorphous calcium phosphate, ACP) to crystalline hydroxyapatite. This mimics the organicmatrix-mediated mineralization of native bone and enables the programmable control of mineralization location, morphology and kinetics on the molecular level [8].

The high modifiability and good biocompatibility of polysaccharides enable them to be designed as multifunctional intelligent carrier. For instance, chitosan/gelatin composite scaffolds offer the physical support as well as the possibility for programmed loading and controlled release of bioactive molecules (e.g., VEGF) [9]. Sequential release or microenvironment responsive release [10] (e.g., pH or enzyme-triggered), these materials could accurately control the coupling process of angiogenesis, innervation, and osteogenesis temporally and spatially, thus directing a more complete and accelerated functional bone reconstruction.

Polysaccharides from natural sources also provide excellent biosafety—the degradation products of such polymers tend to be biocompatible, not generating the acidic microenvironment or chronic inflammation that can result from degrading synthetic polymers [11]. In addition, many polysaccharides such as hyaluronic acid are intrinsic parts of the extracellular matrix and they may be active participants in cell signalling by means of receptor mediated mechanisms (e.g. CD44) thus controlling the host cell behavior (adhesion, proliferation, differentiation and migration). Polysaccharide-based material can therefore establish an appropriate "biological dialog" between the materials and the neighboring tissue; dynamically responding to and encouraging the healing microenvironment, not just being an inert, static physical barrier [12].

Hence, our emphasis in a review entitled "advanced bone tissue engineering" is on understanding and exploiting these specific bio-mimetic, synergistic, and dynamically interactive abilities of the polysaccharide materials. All these abilities converge on one goal, which is to create an active self-healing mechanism that can control the onset of crystallization at the atomic level, controlling cellular behaviours at the cellular scale, and finally to reach a functional remodeling of tissues at the tissue scale. Representative polysaccharides like chitosan, dextran and hyaluronic acid have the following inherent advantages so that such as ion chelation, structure modifiability and bioactive regulation provide new ways to solve the systemic bottlenecks of conventional materials in mineralization kinetics, vascularization, biocompatibility. In this review, we provide a structured summary of molecular mechanisms and recent developments on polysaccharide-regulated biomineralization and discuss how these can be integrated to novel next generation tissue engineering design paradigms, which may pave the way to novel orthopaedic reconstruction techniques.

## 2 Biomineralization

### 2.1 Definition of biomineralization

**Fig. 1:**
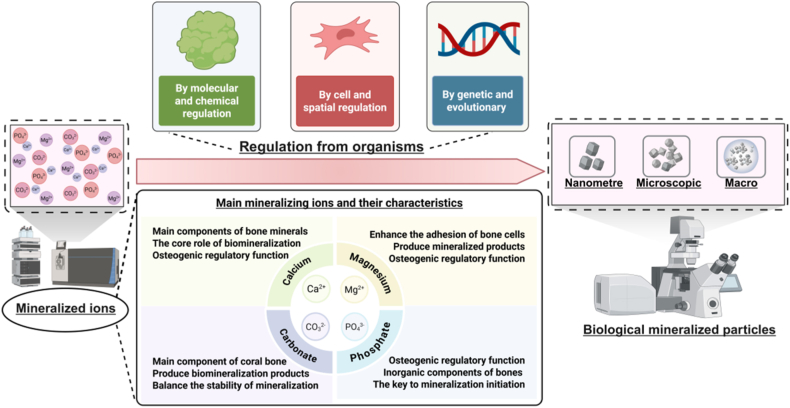
Brief Introduction to Biomineralization. Biomineralization indicates the process by which organisms, through cellular activities, synthesize inorganic materials or organic-inorganic composite materials with well-defined structures in a targeted and controlled manner across macroscopic, microscopic, and nanoscale dimensions. Created with Biorender.

Biomineralization is an ancient biological phenomenon where organisms synthesize inorganic materials or organic-inorganic composites with well-defined structures across macroscopic, microscopic, and nanoscale dimensions through biological processes [13]. Simply stated, it is an organism-mediated process involving the deposition of inorganic materials [14]. In this process, various organisms employ their distinct cellular activities to direct and regulate mineral formation (Fig. 1) [15,16]. Examples include siliceous architectures in diatoms, calcitic deposits in marine invertebrates, and phosphate-carbonate composites in vertebrate bones, each reflecting taxon-specific mineralization patterns [17,18].

### 2.2 Importance of biomineralization

Biomineralization connects living organisms with inorganic entities, offering critical insights for medicine, tissue engineering, materials science, and environmental science, and facilitating interdisciplinary research [[19], [20], [21]].

**Fig. 2:**
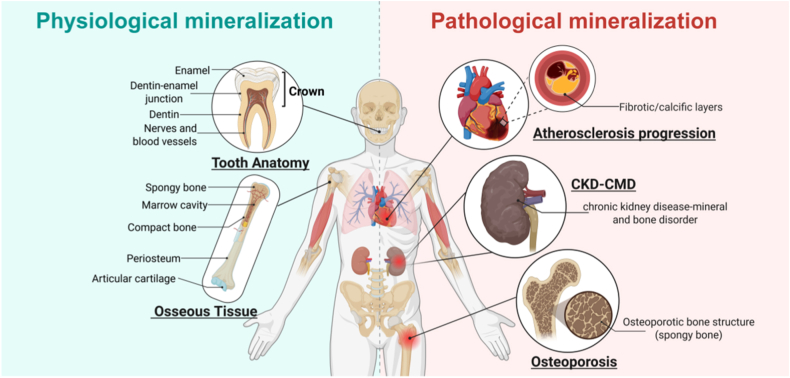
The Comparative Analysis of Physiological and Pathological Mineralization in Biomineralization. Biomineralization can result in either positive or negative outcomes, contingent upon alterations in cellular function and the microenvironment at the biomineralization site. In humans, physiological mineralization primarily occurs in the hypertrophic regions of teeth, bones, and growth plate cartilage. On the other hand, pathological mineralization can take place in any tissue within the human body. Created with Biorender.

It can have positive or negative outcomes based on cellular function and microenvironment. Physiological mineralization occurs in teeth, bones, and growth plate cartilage, while pathological (ectopic) mineralization affects various tissues, demanding effective therapeutic strategies (Fig. 2) [22]. Nowadays, diseases associated with "ectopic mineralization" and bone tissue mineralization deficiencies are highly prevalent, necessitating the urgent identification of effective therapeutic strategies for such conditions [[23], [24], [25], [26]]. The aberrant expression of osteoblast-related markers plays a critical role in diseases associated with "ectopic mineralization," including chronic kidney disease-mineral and bone disorder (CKD-MBD), and generalized arterial calcification of infancy (GACI) [[27], [28], [29]].

Moreover, the Microbially Induced Calcite Precipitation (MICP) discussed earlier promotes the growth of mineral deposits, including calcium carbonate, through the carbonate and calcium ions generated by bacteria and other microorganisms during their metabolic processes [30]. This process effectively immobilizes heavy metal ions in natural environments, such as soil or water, thereby reducing their bioavailability and toxicity [31,32]. Thus, exploring the precise molecular mechanisms of biomeramination holds critical importance and value. This kind of study can not only aid in pinpointing possible therapeutic targets for associated diseases but also offer meaningful perspectives and applicable approaches for environmental restoration and other connected fields.

### 2.3 Categories of biomineralization

Biomineralization is divided into Biologically Induced Mineralization (BIM) and Biologically Controlled Mineralization (BCM), based on the organism's control over the process [14].

**Fig. 3:**
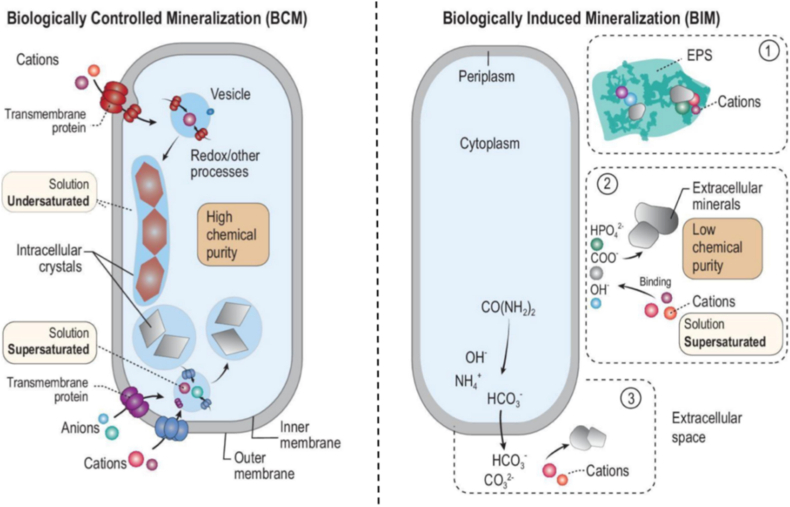
Schematic representation of the mechanisms underlying BCM and BIM. In BCM, anions and cations are actively transported into the cell via transmembrane proteins. Subsequently, biochemical processes governed by genetic regulation lead to supersaturation and precipitation of minerals within the intracellular membrane, despite being in an unsaturated state extracellularly. In BIM, mineral formation occurs through three potential pathways: (1) The interaction of anions and cations within extracellular polymeric substances induces mineral nucleation; (2) Anions present on the cell surface induce mineral nucleation; (3) Extracellular anions interact with cations to facilitate mineral precipitation [33]. Copyright 2022 The Authors.

BIM means a kind of biomineralization mechanism which is triggered indirectly by organisms with the help of their physiology metabolism action, or the change on physicochemistry property of surrounding solution environment. It usually happens while one organism changes its environment with its own metabolism action, resulting in supersaturation and subsequent precipitation of some mineral out-of-cell. The initial step consists of the electrostatic attraction of cationic to anionic functional groups present at the cell surface or in extracellular polymeric substances (EPS). The minerals that are precipitated through BIM, usually have special morphology and can also contain certain types of organic matter (Fig. 3) [33]. In contrast, BCM enables precise regulation of the nucleation, growth, and assembly of minerals by organisms, thereby forming biomimetic mineralized materials with specific functionalities and structures. BCM is strictly governed by a suite of genes that control element/ion influx, intracellular biochemical pathways, and crystal formation within the cell. While the extracellular environment typically remains unsaturated, the intracellular environment becomes supersaturated, facilitating mineral precipitation (Fig. 3) [33,34]. This mineralization process primarily involves the interaction between organic substances, such as polysaccharides and proteins secreted by organisms, and mineral ions. This interaction promotes the formation, stabilization, and phase transition of amorphous mineral precursors at the right location, time, and rate, thus propelling biomineralization [35].

These contrasts between BIM and BCM provide us with an insightful framework for thinking about, and designing, polysaccharide-mediated biomineralization approaches: Whereas BIM focuses on the contributions from the environment and waste products, BCM underscores the exact genetic and molecular control in mineral formation. As for bone tissue Engineering, polysaccharides may be designed to operate in either paradigm, or indeed a combination thereof. For example, a polysaccharide scaffold may simply passively induce mineral supersaturation (BIM-like), but alternatively the scaffold could be functionalized with bioactive molecules in order to actively drive cell-mediated mineralization (BCM-like). In what follows we discuss such approaches, explicitly relating the mechanisms of a particular polysaccharide with either BIM, BCM, or hybrid mode.

### 2.4 Conditions of biomineralization

Biomineralization is governed by the intricate interplay between environmental physical and chemical parameters and the synergistic actions of biomolecules. Within this process, minor variations in pH, temperature, and ion availability, together with the structural templating and regulatory functions of macromolecules (particularly polysaccharides and proteins) dictate the spatial localization, temporal progression, and morphological characteristics of biomineralization [[36], [37], [38], [39], [40], [41], [42]]. Alterations in different environmental conditions can have a substantial impact on the results of biomineralization processes. Suitable environmental factors can improve the development of biominerals. For instance, in an environment with alkaline pH, calcium ions (Ca^2+^) are more likely to interact with carbonate ions (CO_3_^2−^), which causes the deposition of calcium carbonate and supports the process of biomineralization. Appropriate concentrations of key ions (e.g., Ca^2+^, Mg^2+^, CO_3_^2−^, PO_4_^3−^) can facilitate the nucleation and growth of mineralized products, whereas excessively high or low ion concentrations may suppress the process of biomineralization [[43], [44], [45]].

Biomolecules, including polysaccharides and proteins, exhibit multi-dimensional and multi-scale regulatory capabilities during the process of biomineralization, surpassing the simplistic physical template effect. These biomolecules serve as a clearly defined chemical and physical template for mineral nucleation while also regulating the growth direction of minerals. This process ultimately results in the formation of biominerals characterized by specific shapes and structures [[46], [47], [48], [49]]. These biomacromolecules collaborate to achieve precise regulation of mineral nucleation sites, crystal orientation, phase stability, and macroscopic morphology, thereby forming biominerals with hierarchical structures and superior mechanical properties [[50], [51], [52], [53]]. This indicates that polysaccharides and proteins are essential in the biomineralization process.

### 2.5 A comparison between traditional mineralized materials and biomineralized materials

Conventional mineralized materials (e.g., hydroxyapatite, metallic implants) have high stiffness but are chemically inert, impairing cell interactions and native bone structure replication, leading to poor integration and complications [54]. Conversely, biologically induced mineralization employs polysaccharide–protein matrices as programmable scaffolds that orchestrate nucleation, crystal orientation and growth kinetics of the inorganic phase. Bottom-up assembly yields hierarchical, bone-mimetic constructs combining mechanical competence with tunable bioactivity and resorption, thus presenting a "living" material concept for regeneration [51]. The following sections will therefore summarize the properties, recent progress and future refinement of polysaccharide-based biomineralization systems in tissue-engineering design.

## 3 Polysaccharide materials affecting biomineralization

### 3.1 Overview of polysaccharides

Polysaccharides are naturally occurring complex biomolecules with distinctive physicochemical properties and biological activities [55]. Polysaccharides are naturally occurring complex biomolecules with distinctive physicochemical properties and biological activities. As high-molecular-weight carbohydrate polymers, they consist of ≥10 monosaccharide units linked by glycosidic bonds (covalent linkages between adjacent carbons via an oxygen bridge) in living organisms, with structures that may be linear or branched [55,56]. The basic components of polysaccharides are monosaccharide units, with typical instances being glucose, fructose, and galactose. These units are connected through glycosidic bonds, forming chains that can be either branched or linear [57,58].

**Fig. 4:**
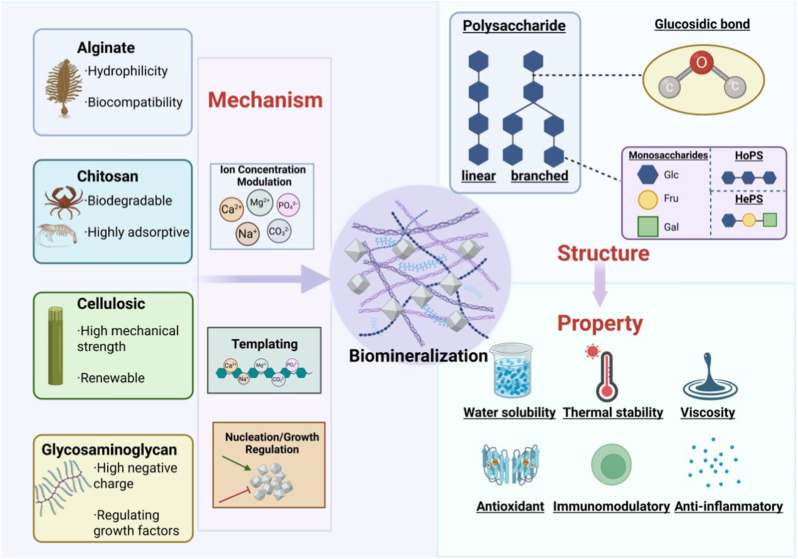
The Characteristics and Mineralization Mechanism of Polysaccharides. This illustration shows diverse natural biopolymers (for example, alginic acid, chitosan, cellulose, and glycosaminoglycans) along with their characteristics. It also meticulously details their action mechanism during the biomineralization process. Through the regulation of ion concentration, templating, and the nucleation and growth mechanisms of crystals, these polymers are able to form either linear or branched polysaccharide architectures. This significantly influences the properties of materials, covering aspects such as water solubility, thermal stability, antioxidant capacity, and immunomodulatory capabilities. Created with biorender.

Polysaccharides can be classified into two main categories: homopolysaccharides (HoPS) and heteropolysaccharides (HePS), depending on the types of monosaccharides they comprise. Homopolysaccharides are made up entirely of a single type of monosaccharide unit, with typical examples being starch and cellulose. On the other hand, heteropolysaccharides contain a variety of different monosaccharide units, including compounds like hyaluronic acid and agar. These structural distinctions between the two groups lead to differences in their physicochemical characteristics and biological functions [[59], [60], [61]]. Polysaccharides exhibit complex structures that extend beyond primary configurations to include secondary, tertiary, and even quaternary structures. Their functional roles and biological activities are intricately linked to their spatial conformations (Fig. 4) [[62], [63], [64], [65]].

Polysaccharides are widely distributed in nature and are essential building blocks of many organisms such as plant, animals and microorganisms. Starch and cellulose are usually found in plant. Glycogen and other polysaccharides are usually found in animal. Microorganism can also produce an amazing variety of polysaccharides such as chitosan and hyaluronic acid [[66], [67], [68]]. In the next sections we will deeply discuss the main polysaccharides involved in the biomineralization process focusing especially on chitosan and hyaluronic acid.

Polysaccharides are essential in natural and medical biology because of their numerous structures and various functions [[69], [70], [71]]. In this part, we briefly discuss the structure characteristic, physicchemical properties, and biological functions of polysaccharides.

#### 3.1.1 Characteristics of polysaccharide

The structure properties of the polysaccharides mainly include the composition of monosaccharides, the type of linkages, degree of branching and molecular weight. There are complex structures of polysaccharides, which are linear or branched. The structure space and molecule weight both affect polysaccharides functions [[72], [73], [74], [75]].

The polysaccharide structure is mainly controlled by three factors: (1) the diversity of mono-saccharide structure, (2) regio-specificity and anomericality of glycosidic bonds, (3) the linkage sequence-dependent chain connectivity determining the chain structures. Polysaccharide chain bind together through hydrogen bonding which results in a network of hydrogen bonding making up the secondary structure of polysaccharides [76]. For instance, the well-known parallel chain crystal structure of cellulose and the helical conformation of amylopectin exemplify this concept [77,78]. The hydroxyl, carboxyl, and other functional groups on the sugar chain are capable of interacting via non-covalent bonds. This interaction leads to the formation of specific three-dimensional structures, such as triple helix configurations, which characterize the tertiary structure of polysaccharides. In addition, multiple sugar chains can interact via non-covalent bonds to form aggregates with complex spatial arrangements, such as those observed in the crystalline regions of starch, referred to as quaternary structures of polysaccharides [[78], [79], [80]]. Structural proprieties of the polysaccharides bestow them the strength, hydrogen bonds providing a wood-like resilience. In addition, remarkable mechanical strength variations between polysaccharides and isopolysaccharides have been found [81,82]. Research into the structural characteristics of polysaccharides provides both theoretical support and experimental reference for creating superior materials from polysaccharides.

#### 3.1.2 Physicochemical properties of polysaccharides

As the physicochemical properties of polysaccharides are closely associated with their structural characteristics and act as an external reflection of structural characteristics. Polysaccharides are diverse in the physicochemical properties. Dissolution of polysaccharides in water is one of the most important physicochemical properties of polysaccharides, which is related to both molecular structure and molecular weight. The polysaccharides e.g., pectin and dextran with a high amount of hydrophilic groups display a greater water solubility compared to the cellulose with a highly crystalline structure and a strong intermolecular hydrogen bonding. This is essentially due to the relative high number of hydrophilic and hydrophobic groups of the polysaccharides and different inter- and intramolecular hydrogen bond [[83], [84], [85]]. Viscosity, a key physicochemical property of polysaccharides, is regulated by molecular weight, side-chain topology, and the type and position of glycosidic linkages [86,87]. Thermal stability also varies with structural features [88,89]. For the design of polysaccharide-based biomineralizing materials, evaluating the compatibility of the selected polysaccharides' physicochemical properties with application requirements is critical.

#### 3.1.3 Biological activity of polysaccharide

**Fig. 5:**
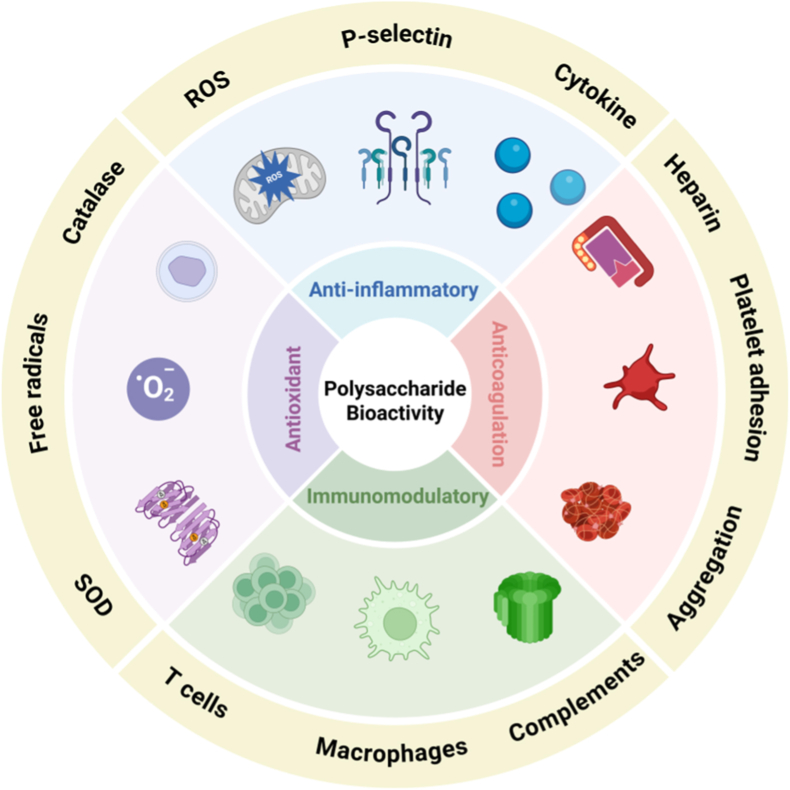
Bioactivities of polysaccharides (anticoagulation, antitumor, antioxidant, anti-inflammatory and immunomodulatory). A diagram of the various bioactivities of polysaccharides, such as antioxidant, anti-inflammatory, immunomodulatory, and anticoagulation. For the antioxidant activity, it involves the following antioxidants, free radical, superoxide dismutase (SOD), catalase. The anti-inflammatory action is related to reactive oxygen species (ROS), cytokines, Pselectin, respectively. The immunomodulating action is related to immune cell and factor such as T cells, macrophages, complements, respectively. The anticoagulant action is related to heparin, platelet adhesion and aggregation respectively. These bioactivities are interrelated by the action of polysaccharides collectively showing their combined and varied biological roles. Created with biorender.

The biological functions of polysaccharides are revealed through their multifaceted and vital roles in organisms (Fig. 5). Polysaccharides adjust immune responses by triggering various antigen-presenting cells through pattern recognition receptors. Specifically, the degree of sulfation and the extent of branching in polysaccharides play key roles in determining the efficiency of immune regulation [90,91]. Furthermore, polysaccharides exhibit antioxidant capabilities by removing reactive oxygen species such as hydroxyl radicals and superoxide anions. They also boost the effectiveness of the body's inherent antioxidant systems, thereby offering protective advantages [92,93]. Polysaccharides anti-inflammatory effect *in vivo*, reduce inflammation by inhibiting the synthesis and secretion of inflammatory factors by regulating the pathways of anti-inflammatory signaling [94,95]. In addition, polysaccharides show various biological activities, such as anti-cancer, antiviral and anticlotting. The different biological activities mentioned above reflect the ability of polysaccharides to address several health ailments.

### 3.2 Polysaccharide selection affecting biomineralization

Biomineralization refers to the process of mineral deposition in biological systems, which involves the synergistic interaction of multiple biomolecules. Different polysaccharides contribute uniquely to biomineralization by regulating mineral formation through three mechanisms: acting as templates, modulating mineral ion concentrations, and bidirectionally regulating the nucleation and growth of specific minerals (promotion or inhibition). Commonly studied polysaccharides, including glycosaminoglycans, alginates, chitosan, and cellulose, regulate biomineralization through their unique physical structures and physicochemical properties.

#### 3.2.1 Alginate

Alginates belong to a category of common polysaccharides that are extensively found in land plants and marine creatures. The distinctive characteristics of alginate enable it to hold a significant position in biomineralization procedures. Acting as a hydrophilic anionic polysaccharide with outstanding biocompatibility, alginate can form physical gels when exposed to divalent cations [96]. It mainly consists of α-L-mannuronic acid (Man) and α-D-glucuronic acid (Glu) [97,98]. Alginate possesses a high density of negative charges, conferring excellent solubility in aqueous environments. Moreover, alginate possesses several beneficial characteristics, including the ability to degrade naturally in biological environments, compatibility with living tissues, and minimal immune response induction. The alginate solution in saline exhibits non-Newtonian fluid properties, where its thickness rises with decreasing pH, peaking between pH levels of 3.0 and 3.5. The pH-dependent behavior of alginate originates from carboxyl moieties distributed within its polymer structure. Under alkaline conditions, this polysaccharide demonstrates enhanced hydration capacity through volumetric expansion, thereby emphasizing its pronounced responsiveness to environmental pH variations [99]. We can extract natural alginate from brown algae. Alginate exhibits excellent biocompatibility, biodegradability, and gel-forming capability [100]. It can also influence biomineralization by providing templates and specific sites for mineral deposition [101].

#### 3.2.2 Chitosan

Chitosan is a bio-derived polysaccharide, the chemical name is (C_6_H_11_NO_4_) and possesses the characteristics of biocompatibility, biodegradability, and high adsorption efficacy. It is also low cost, safe, nontoxic, has a variety of derivatives and so on. All these merits make it to be a highly demanded raw material in various areas of medical treatments [102]. It has been reported that chitosan can partake in biomineralization process by imitating the part of the role of bacterial extracellular polymeric substances that associated with biomineralization [103]. Moreover, chitosan can promote the osteoblast proliferation and adhesion in human body and strengthen the bone-related bone formation by osteoblasts, thus promoting mineralization process of physiologic action [104].

#### 3.2.3 Cellulosic

As a biopolymer with extensive sources, rich surface chemical, good biocompatibility, mechanical strength, renewability, cellulose is an excellent biological template for the inorganic component biomineralization [105]. Cellulose promotes mineralization *in vivo*, bone defect repair, bone microstructure refinement has been demonstrated in research. In addition, cellulose enhances the biomechanical performance of bone tissue by modulating gene and protein expression involved in bone homeostasis. These results highlight the potential utilization of cellulose-based polysaccharides to biomimetic systems for biomineralization [106].

#### 3.2.4 Glycosaminoglycan

Glycosaminoglycan is a broad type of negatively charged polysaccharide that widely distributes in the extracellular matrix. Researches have showed that glycoginominoglycan could control the local ion concentration by binding to calcium ions and the other minerals by the negatively charged groups, and the mineral deposition could be affected by controlling the biomineralization process.

Hyaluronic acid, as a component of glycosaminoglycans, represents a class of polysaccharide molecules that exhibit high biocompatibility and biodegradability [107]. It promotes biomineralization by establishing a favorable physical environment, which is achieved through the creation of a gel-like structure by binding a large number of water molecules [108].

Chondroitin sulfate (a sulfated glycosaminoglycan) accelerates the Ca^2+^ ions’ accretion on collagen fibrils and prominently enhances the collagen mineralization. In terms of mineralization, chondroitin sulfate has been found to directly activate collagen matrix mineralization, accelerate the mineral deposition, and induce biomimetic mineralization [109,110]. Moreover, heparan sulfate is a highly sulfated glycosaminoglycan, able to bind to broad categories of growth factors and minerals [111].

While each of these polysaccharides contributes to biomineralization, their underlying mechanisms, driven by distinct chemical structures and physicochemical properties, vary significantly. These differences directly influence their efficiency, application scenarios, and suitability for bone tissue engineering. To provide a clear and comparative synthesis of their biomineralization mechanisms, key characteristics, and engineering implications, we have summarized the salient features of several representative polysaccharides in (Table 1). This research not only provides rich knowledge of the biomineralization, but also provide a good theoretic basis to fabricate new biomaterials.

**Table 1:** Distinct biomineralization mechanisms of representative polysaccharides.

Polysaccharide Type	Key Functional Groups	Main Biomineralization Mechanisms	Key Impacts on Mineralization	Citation
Alginate	Carboxyl (–COOH)	1. Template for mineral deposition: Forms ionic gels with Ca^2+^, providing confined spaces for nucleation and growth. 2. Specific deposition sites: Ca^2+^ bound in gels acts as local source for mineral deposition. 3. pH-responsive environment: Modulates ion solubility and precipitation kinetics.	Promotes fine and uniform mineral deposition within gel matrices.	[96,97,99]
Chitosan	Amino (–NH_2_)	1. Nucleation template: Functional groups (–NH_2_, –OH) provide active sites for hydroxyapatite nucleation, mimicking extracellular polymeric substances. 2. Bioactivity promotion: Enhances osteoblast adhesion and activity, creating a favorable biological environment for mineralization.	Promotes formation of carbonated hydroxyapatite layers with bone-like composition.	[103,104]
Cellulose	Hydroxyl (–OH)	1. Biological template for inorganic component mineralization. 2. Regulates bone homeostasis-related gene/protein expression.	Promotes *in vivo* mineralization, bone defect repair, optimizes bone microstructure, and enhances biomechanical properties.	[105,106]
Hyaluronic Acid	Carboxyl, hydroxyl, acetyl groups	1. Hydrogel microenvironment regulation: Forms highly hydrated gel-like matrices that regulate diffusion of ions and nutrients.	Creates a stable hydrated mineralization microenvironment, facilitating uniform dispersion and oriented deposition of mineral particles.	[[109], [110], [111]]
Chondroitin Sulfate	Sulfate (–OSO_3_^-^)	1. Strong ion binding: Rapidly binds Ca^2+^, significantly increasing local calcium concentration. 2. Activates collagen matrix mineralization.	Accelerates calcium ion accumulation and enhances collagen mineralization.	[107,108]

This table systematically compares five representative polysaccharides in terms of their key functional groups, main biomineralization mechanisms, and key impacts on mineralization. Alginate primarily acts as a template and provides pH-responsive deposition sites, promoting fine mineral deposition. Chitosan mimics extracellular polymers and enhances osteoblast activity, leading to bone-like hydroxyapatite formation. Cellulose serves as a biological template and regulates bone-related gene expression, facilitating *in vivo* mineralization and bone repair. Hyaluronic acid constructs a hydrated gel microenvironment to enable uniform mineral dispersion. Chondroitin sulfate strongly binds Ca^2+^ and activates collagen mineralization, accelerating calcium accumulation and collagen mineralization.

### 3.3 Chemical modification of polysaccharides

Although natural polysaccharides have the advantages of good biocompatibility and diverse structures for biomineralization, they also suffer from poor mechanical strength, the control over mineralization kinetics, and cell-specific recognition. To break through those bottlenecks, chemical modification is an important approach to finely tune polysaccharides’ properties and achieve effective biomimetic mineralization. To control their charges, we can modify with particular moieties and attach inorganic materials on the polysaccharide framework, crosslinked network structures, bioactivity, and mineralization guidance. Herein, we summarize the four main strategies of polysaccharide modification and elaborate on their molecular design principles, structure–function relations, and synergy of mechanism in biomineralization.

#### 3.3.1 Charge-regulating modifications

The main charge-regulating modifications for polysaccharides are sulfation and carboxylation. They aim to change in a controlled way the charge density and its position on the polysaccharide chain, thus accurately controlling interactions with mineralization ions like calcium or phosphate, and therefore affecting the nucleation, growth and assembly processes in biomineralization.

Sulfate (–OSO_3_^–^) groups introduced in the polysaccharide backbone, as well as an increase in net negative surface charge; directly participates in binding and nucleating mineral ions [112,113]. It has been found that controlled chemical sulfation on natural alginate could generate alginate derivatives (ASs) with various degrees of sulfation (DS) and sulfation pattern [114]. And within a certain extent, a higher DS corresponds to higher mineralization efficiency.

Carboxylation modification is to introduce carboxylic acid groups (–COOH) on polysaccharide, which can increase the negative charge and metal ions coordination capacity of polysaccharide. Its main mechanisms are as follows: provide an ordered site for nucleation by forming a stable coordination center with Ca^2+^ [103], as well as influencing adsorption and growth of mineral precursors through increased negative charge density, and altered chain conformation [115]. For example, carboxymethyl chitosan (CMCS), with an efficiency on the calcium precipitation equal to 96.07 % for a concentration of 0.15 %, much more than that of non-modified chitosan with nucleation based on multiligand coordination between Ca^2+^ and -NH_2_, -OH, -COOH groups [103]. Also, carboxylated agarose complexed with silk fibroin can direct formation of sheet-like and rod-like hierarchical structures of hydroxyapatite, simulating the ordered assembly of natural biominerals [116].

In conclusion, the processes of sulfation and carboxylation contribute two key aspects: an increase in negative charge density and an augmented ability to coordinate metal ions. This enables systematic control over the electrostatic properties and three-dimensional functionality of polysaccharides. Such molecular-level modifications offer precise strategies for the targeted engineering of high-performance biomineralization materials.

#### 3.3.2 Introduction of reactive group modifications

Reactive group (e.g., methacrylation), which is introduced into the polysaccharide chain through covalent grafting of photopolymerizable methacrylate groups (-MA). Under irradiation with a photoinitiator at a certain wavelength, the-MAs are rapidly and controllably covalently crosslinked into structurally stable and precisely shaped 3D networks. Such networks offer a spatial confinement to the heterogenous nucleation/growth of minerals, but also dramatically improve the mechanical properties as well as the dimension stability of the base material [117].

In practice, the polysaccharides of different sources which are modified by methacrylation have good effects on guiding minerals. For example, methacrylated gellan gum (GG-MA) [118], after being pre-crosslinked using CaCl_2_ into calcium rich microspheres, could spontaneously cause a bone-like apatite deposition in simulated body fluid; this mineral phase was very biomimetic in terms of composition and structure, suggesting their great potentials in bone tissue engineering. In the same way, κ-carrageenan efficiently methacrylated by microwaves shows good photocurability characteristics [119], and hydrogels based on it are able to sustain chondrocytes growth, differentiation, and extracellular matrix mineralization, thus being applicable to the use of cartilage repair and regeneration.

In conclusion, the methacrylation process employs a combination of mechanisms, such as photo-induced shaping and ionic affinity, to convert inert polysaccharides into smart templates. These templates are capable of actively and precisely directing the process of biomineralization. This methodology offers a highly adaptable and robust molecular tool for the creation of biomimetic mineralization materials, which possess both pre-designed structures and functions.

#### 3.3.3 Biomimetic functionalization modifications

Polysaccharide biomimetic functionalization is aimed at constructing a bioactive, structurally supportive microenvironment through mimicry of the chemical composition and structural features of the native extracellular matrix (ECM). The principle is based on incorporation of bone morphogenic protein (BMP) and arginine glycine aspartic acid (RGD) sequence, which modulate the signaling pathways involved in osteogenic differentiation and cell–material interfacial interactions, respectively, thereby synergically promote the biomineralization process.

RGD is one of the major cell adhesion recognition sequences found on ECM proteins and binds selectively with cell surface integrin receptors (e.g.αvβ3, α5β1), which activates the intracellular signaling cascade of focal adhesion kinase (FAK) to promote cell spreading and proliferation, setting up a stage of the following osteogenic differentiation [120]. For example, Wang et al. [121] employed layer by layer self-assembly to grafted glycine-arginine-glycine-aspartate-serine (GRGDS, one kind of RGD sequences) peptide on chitosan/oxidized alginate composite membrane surface greatly enhancing the adhesion and proliferation efficiency of bone marrow mesenchymal stem cell (BMSC). Huang et al. [122] introduced the Glycine-Arginine-Glycine-Aspartic Acid-Serine-Proline-Cysteine (GRGDSPC) peptide into a collagen/hyaluronic acid composite coating via disulfide crosslinking, which significantly increased the expression of key osteogenic genes such as Runx2 and osteocalin in MC3T3-E1 cells and augmented mineralized nodule area. Similarly, Brun et al. [123] covalently grafted the Glycine-Arginine-Glycine-AsparticAcid-Serine-Proline-Lysine (GRGDSPK) peptide onto chitosan, substantially improving the adhesion and proliferation rates of human osteoblasts compared to unmodified chitosan.

Regarding the BMP-mediated osteoinductive mechanism, BMP (particularly BMP-2), as a potent osteoinductive factor, primarily promotes the differentiation of mesenchymal stem cells into osteoblasts [124]. Polysaccharide-based carriers can achieve sustained release and protection of BMP bioactivity through methods such as covalent conjugation or nanoencapsulation. For instance, Jeon et al. [125]developed a heparin-alginate photo-crosslinked hydrogel that utilized the affinity interaction between heparin and BMP-2 to effectively prevent burst release, significantly enhancing bone formation and calcium deposition in ectopic osteogenesis experiments.

The synergistic effect of RGD and BMP is a key advantage of polysaccharide biomimetic modification in promoting biomineralization. The two form complementary regulatory networks from the dimensions of cell adhesion and differentiation signals, respectively. Wang et al. [121] constructed a biomimetic ECM coating co-loaded with GRGDS and BMP-2, enhanced the alkaline phosphatase activity of BMSCs in vitro. These studies fully demonstrate that polysaccharide carriers, by synergistically integrating the adhesive guidance of RGD and the osteo-inductive function of BMP, can construct a biomimetic microenvironment that efficiently promotes biomineralization.

#### 3.3.4 Inorganic phase hybridization modifications

The inorganic phase hybridization introduces additional ingredients such as nano-hydroxyapatite (nHA) and bioactive glass (BG) into the system to supply a stable heterogeneous nucleation site for the polysaccharide matrix. It regulates local ion concentration and spatial distribution, this approach can direct and accelerate bone-like apatite deposition [126].

Taking bioactive glass for instance, it is possible to increase the bioactivity and osteo-inductive ability of scaffolds by combining them with polysaccharides. The incorporation of BG in a gelatin-alginate composite scaffold could improve both the porosity and the mechanical properties of the material while allowing the controlled release of Si^4+^, Ca^2+^, and PO_4_^3−^ in a simulated body fluid solution. This leads to the enrichment and induction of a well-crystallized hydroxyapatite layer on the material surface which significantly increases the alkaline phosphatase (ALP) activity of osteoblasts and promotes calcium nodule deposition [126]. In another study it was also proven that vanillin cross-linked chitosan/BG scaffolds showed uniform calcium phosphate deposits on its surface after immersion, with a Ca/P atomic ratio equal to 1.64, close to that of natural bone mineral, further showing how effective BG is for inducing a biomimetic mineralization [127].

For the hydroxyapatite hybridization system, mixing of nHA and polysaccharides may imitate a similar mineral component to that in natural bone, transferring good osteo-conductivity to the material. An injectable hydrogel fabricated from blending nHA and sodium alginate directed new bone growth in a rabbit bone defect model, including mature Haversian system, primary osteons and a collagen fiber-rich and HA crystalriched organic matrix [128]. Similarly, the incorporation of nHA in to a carboxymethyl chitosan reinforced composite hydrogel scaffold could produce a hierarchical microporous structure, providing a suitable microenvironment for cell adhesion, proliferation and mineralization [129].

Hence, the inorganic hybridization of polysaccharides to BG or HA greatly increases their ability for biomineralization and bone regeneration via several means, including acting as a nucleation site, modulating the ionic microenvironment and enhancing the mechanical properties.

To sum up, chemical modifications like sulfation/carboxylation, methacrylation, biomimetic peptides/growth factors grafting, and inorganic phase hybridization could improve the biomineralization ability of polysaccharide material systemically from different angles, including the charge features, cross-linked network, biological recognition or nucleation templates. In addition to mimicking the synergetic interaction of organic and inorganic components in nature, these strategies offer an abundant pool of molecules as well as design principles for creating next generation bone repairing materials that possess active mineralizing inducing function.

## 4 The role of polysaccharides in biomineralization and their applications in bone tissue engineering

Biomineralization is fundamental to osseous tissue regeneration strategies. Polysaccharides have a crucial regulatory function in coordinating this mineralization process. As a result, in bone tissue engineering for efficient bone healing, researchers often use polysaccharides to regulate biomineralization.

### 4.1 Chitosan

The chitosan, on account of its multiform bioactive sites like amino, aldehyde, carboxyl, etc., plays an active role by interacting with calcium and phosphate ions to serve as a natural nucleation sites to start the beginning of biomineralization process of forming hydroxyapatite [130]. Zhong et al. developed a chitosan maleate/PEGDA hybrid hydrogel through photopolymerization and promoted the in vitro formation of carbonated.

Chitosan has been widely used in bone tissue engineering because of its excellent biocompatibility and good cellular interaction. It is considered an ideal scaffold material, playing an important role in promoting biomineralization *in vivo* [131]. Nguyen et al. synthesized three-dimensional chitosan/hydroxyapatite (CS/HAp) biomimetic scaffold via freeze-drying method. Scaffold demonstrated appropriate pore structure, swelling degree, tensile strength and biodegradability. After immersing in simulated body fluid (SBF) for 15 days, a bone-like apatite layer was observed on the CS/HAp scaffold surface which indicates its excellent bio-mimetic mineralization performance. This could imply good potential application in bone tissue engineering. Saiedeh et al. combined 3D printing and electrospinning methods to create a polylactide/polyethylene glycol/Brady stone (PLA/PEG/B) scaffold coated with chitosan-polyethylene glycol (CS-PEG) nanofibers. This structure incorporated vancomycin (V) and insulin-like growth factor-1 (IGF-1). The resulting composite could show better bacteria-killing ability, enhanced biocompatibility, increased bone formation, and could serve as a candidate for filling bone defects [132] hydroxyapatite by employing a modified simulated body fluid mineralization approach.

Chitosan possesses multiple bioactive moieties, such as amino, aldehyde, and carboxyl groups. These functional units actively engage with calcium and phosphate ions, establishing foundational nucleation sites critical for biomineralization processes. As a result, they facilitate the development of hydroxyapatite [130]. Zhong et al. developed a chitosan maleate/PEGDA hybrid hydrogel through photopolymerization and promoted the in vitro formation of carbonated hydroxyapatite by employing a modified simulated body fluid mineralization approach. These experimental findings demonstrated that the hydrogel's pore size decreased significantly as a result of direct mineral deposition on the pore walls during the mineralization process, while the mineral content increased with prolonged mineralization time. This hydrogel, based on an acidic polysaccharide, functioned not only as a location for mineral binding reactions but also played a role in stabilizing the amorphous inorganic component during the initial mineralization phase. As a result, it successfully controlled the shape, dimensions, and crystalline structure of the minerals [133]. Saba et al. created a composite hydrogel exhibiting osteogenic characteristics by mixing N-carboxyethyl chitosan (CEC), hyaluronic acid aldehyde (HA-ALD), hydrazine adipate (ADH), and sodium alginate (ALG). Saba et al. prepared a combination of hydrogel with an osteogenic function composed of CEC and HA-ALD crosslinked by ADH and ALG in addition to BG particle. After being immersed in simulated body fluid the product released HA–particles. It has also been reported that this hydrogel is able to be healed by itself, containing dual crosslinking feature, and have biological activity that favors cells growth and mineralization. This fact makes this hydrogel a great perspective formulation for ink used for 3D printed hydrogel [134].

**Fig. 6:**
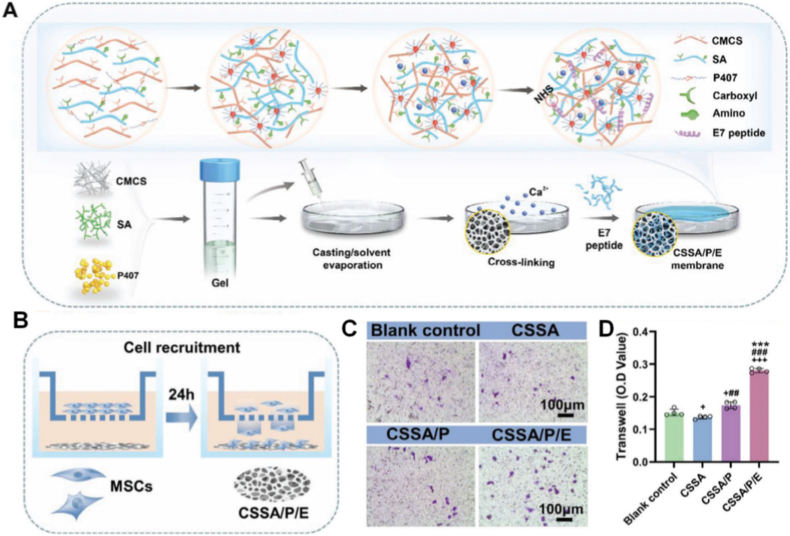
Fabrication and MSCs recruitment ability of the composite membranes in vitro. A) Illustration of the process for fabricating the composite membranes. B) Schematic illustration of the composite membrane attracting MSCs. Transwell-migration images C) and quantitative assessments D) for MSCs attracted by the composite membranes and the control surface following 24 h of incubation [137]. Copyright 2025 Advanced Science.

As a key component in film preparation, chitosan can widely use [135], but standalone chitosan is likely to be limited in the ability to form films due to the low mechanical strength and processability [136]. Thus, researchers often adopt ways of compounding or chemical modification to improve its performance. Li et al. prepared an electrostatically-interacting/cross linked CaCl_2_-complex membrane of CMCS and SA with a microporous structure. They further optimized its microporous structure by mixing the PM with Poloxamer 407 (P407), then linked the E7 peptide to it via N-Hydroxysuccinimide (NHS) cross linking chemistry to prepare a functionalized membrane as CSSA/P/E. The membrane possesses a uniform microporous structure with pore size of about 2.5 μm and porosity is 41.40 %. At the same time, it also has high mechanical strength, super hydrophilicity, and degradability. The experimental study showed that the synergistic effect of microporous structure and E7 peptide modification remarkably enhanced the biological activity of periosteum. The proposed method offers a cell-free, growth factor-free approach to the treatment of large bone defects in clinic settings (Fig. 6) [137].

The blending of chitosan and gelatin improves cell attachment, growth, and movement, all of which are essential for bone regeneration as they constitute the fundamental processes of bone tissue development. Xu et al. utilized α-arbutin (ARB) to cross-link chitosan (C) and gelatin (G), thereby preparing an antioxidant film (CG-ARB). The CG-ARB film was observed to have a uniform surface that became increasingly smooth and compact following the cross-linking process. Furthermore, both the swelling and degradation rates were substantially decreased, suggesting its potential as an effective material for addressing age-related bone conditions [138].

Burcu et al. utilized the electrophoretic deposition (EPD) technique to uniformly deposit chitosan nanoparticles/microspheres onto the titanium surface. By optimizing the deposition parameters, such as voltage (20 V) and time (3 min), they successfully prepared a chitosan coating. This coating substantially enhanced the mineralization capability of the titanium substrate, with mineralized nodules forming earlier in experimental observations. Moreover, chitosan coating in combination with vitamin D functionalization manifested the synergistic effect effectively improve the biocompatibility of titanium-based materials, which offers a novel method of titanium implant materials surface modification [139].

Chitosan can combine with hydrogel to improve the biocompatibility and capacity to repair the tissue of hydrogels [140]. But pure hydrogel generally does not have enough mechanical strength to meet application, so chitosan can strengthen them. Cao et al. cross-linked N-carboxyethyl chitosan (NCEC) with oxidized dextran (ODex), nano-hydroxyapatite (nHAP) through the formation of a Schiff base. The experimental results showed that the hydrogel prepared by NCEC3/ODex3 gelled very quickly, with a 35-s gelation time, by a porous connected network and with the self-recovering effect, making it desirable for injectable usage, after adding nHAP, the hydrogel could still maintain good biocompatibility and rheology and showed good biomineralization capacity [141].

The excellent biocompatibility, biodegradability and hydrogel forming ability of chitosan make it a good carrier for the delivery of exosome. It promotes exosome release in a controlled manner and also avoids their quick degradation effectively in tissue, which promotes the bone formation [142]. Kang et al. fabricated a 3D hybrid scaffold termed dGQH composed of decellularized extracellular matrix (dECM), gelatin (Gel), quaternary ammonium chitosan (QCS) and nHAp via extrusion-based 3D bioprinting.

Additionally, they isolated exosomes from human adipose-derived stem cells (ADSCs), which were further added to the dGQH scaffold via electrostatic coupling. This resulted in an increase in cell attachment and growth for the loaded exosomes on the scaffold. In addition, the dGQH@Exo scaffold revealed significantly more cell potential in terms of bone forming and blood vessel forming in the lab and living organism experiments [143]. Man et al., reported the use of two biopolymers obtained from natural materials (chitosan and type I collagen), to control EV release kinetics by controlling their relative composition ratios. Gels of EV-loaded chitosan/collagen supported a concentration dependent mineralization of extracellular matrix produced by human bone marrow mesenchymal stem cells (hBMSCs) (increased evocalcium deposition 2.3-fold (p < 0.01) compared to the non-EV containing gels). The thermosensitive chitosan-collagen hydrogel can be of interest as an emerging cell-free tool to facilitate the therapeutic benefits of osteoblast derived EVs in bone regeneration strategies [144].

Chitosan, with its abundant bioactive sites, excellent biocompatibility and modifiable flexibility, has become a core natural polysaccharide material for regulating bone tissue mineralization. The following table systematically summarizes representative studies in recent years, visually presenting the design logic and application potential of chitosan-based materials in bone tissue engineering (Table 2). Such materials are built through multivariate combination of hydroxyapatite, gelatin, bioactive glass and others, paired with proper preparation approaches including photopolymerization, freeze drying, and 3D printing. Their derived materials can closely match the requirements of bone mineralization induction, bone defect repair, implant surface modification in a variety of situations. All these researches prove that the essence of chitosan materials is "designable structure, customizable function". By designing, the entire research and development process for these materials, from preparation strategy to applications is shown in clear way in below Table, providing an organized reference to guide future materials invention (Table 2).

**Table 2:** Recent research on the use of chitosan in the biomineralization of bone tissue.

Material Combination	Preparation Method	Characteristics	Application Scenario	Citation
Maleic acid chitosan/PEGDA hydrogel	Photopolymerization	Pore size decreases during mineralization, mineral content increases over time	Bone mineralization	Zhong et al. [133].
N-carboxyethyl chitosan, hyaluronic acid aldehyde, adipic acid hydrazide, sodium alginate, bioactive glass BG	Mixing	Self-healing and dual-crosslinking properties, high bioactivity	Bone tissue engineering	Saba et al. [134]
Chitosan/hydroxyapatite	Freeze-drying	Suitable pore structure, good swelling ratio, tensile strength, and biodegradability	Bone tissue engineering	Nguyen et al. [145]
Chitosan-polyethylene glycol nanofiber-coated polylactic acid/polyethylene glycol/breidite scaffold	3D printing + electrospinning	Good antibacterial properties and biocompatibility	Bone defect repair	Saiedeh et al. [132]
Carboxymethyl chitosan and sodium alginate, Poloxam 407, NHS crosslinked E7 peptide	Electrostatic interaction and CaCl_2_ crosslinking	Uniform microporous structure, good mechanical properties, hydrophilicity, and biodegradability	Periosteum repair	Li et al. [137]
Chitosan and gelatin + α-arbutin	Crosslinking	Smooth and dense surface, reduced swelling and degradation rates	Drug delivery system	Xu et al. [138]
Chitosan nanospheres/microspheres + titanium material	Electrophoretic deposition	Strong mineralization ability, high bioactivity	Surface modification of titanium-based implant materials	Burcu et al. [139]
N-carboxyethyl chitosan, oxidized dextran, and nano-hydroxyapatite	Schiff base crosslinking	Rapid gelation, porous structure, self-healing ability	Bone mineralization	Cao et al. [141]
Decellularized extracellular matrix, gelatin, quaternized chitosan, nano-hydroxyapatite	3D bioprinting	Rich microchannel network, promotes cell adhesion and proliferation	Bone and vascular regeneration	Kang et al. [143]

### 4.2 Alginate

Alginate is a natural polysaccharide extracted from brown algae, whose molecular structure is rich in carboxyl groups that can effectively bind to metal ions such as calcium ions. This interaction promotes mineral deposition and crystallization, representing the key mechanism by which alginate achieves biomineralization. Due to its high carboxyl group density, alginate primarily functions through a biologically induced mineralization-like mechanism: by chelating calcium ions in the solution, it creates a localized supersaturated microenvironment, thereby inducing the passive deposition of hydroxyapatite. This process aligns with the principle of BIM-like, which drives mineralization by regulating the ionic microenvironment [146]. Through interaction with the ECM, alginate replicates the microenvironment of natural bone tissue, which in turn promotes the *in vivo* biomineralization process [147].

Alginate exhibits a broad application potential in bone tissue engineering due to its excellent biocompatibility [148], ease of molding, and tunable structure, and porosity [149]. During the process of biomineralization, inorganic minerals can be incorporated into alginate-based saline gels, thereby enhancing their mechanical properties and biological activity. This modification renders the gels more analogous to the compositional and structural characteristics of native bone tissue [150,151]. Diaz-Rodriguez's research group pioneered a bioactive bone regeneration scaffold through the integration of marine-derived calcium carbonate particulates (sourced from crustacean exoskeleton byproducts) within sodium alginate hydrogel networks, demonstrating sustainable biomaterial design principles. The findings demonstrated that combining alginate with calcium carbonate particles derived from oysters (OY) substantially enhanced the compressive strength of the hydrogel. Furthermore, this combined approach markedly enhanced ECM mineralization while concurrently augmenting osteogenic differentiation in mesenchymal stem cells (MSCs). Alginate can be shaped into several scaffold shapes (hydrogels and porous scaffolds) which have parameters to be adjusted according to the use, such as design, flexibility, pore structure etc. These modifications may subsequently impact on cell attachment, proliferation, and differentiation [149]. Shi et al. prepared a gelatin-based organic–inorganic composite material (called GA-HA), via a sequential freeze–thaw process and gelatin, sodium alginate, and nHA, as the basic components of the material. A strong porous gelatin with a double-network was built by EDC/NHS chemical cross-linking and Ca^2+^ ion cross-linking released by nHA. The GA-HA hydrogel displayed super cytocompatibility and strong advantage cell attachment, cell growth promotion and bone-forming differentiation capacity in MC3T3-E1 pre-osteoblast [152].

**Fig. 7:**
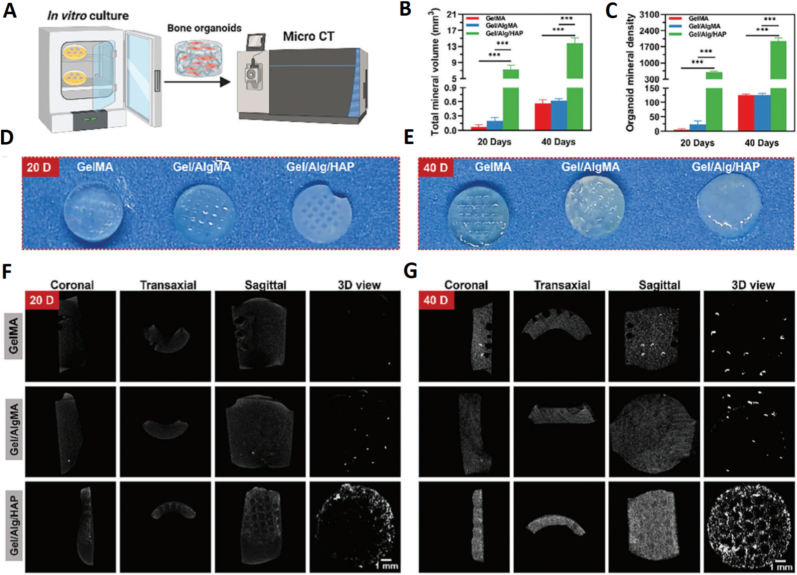
The self-mineralization of gelatin methacrylate (GelMA), GelMA/alginate methacrylate (GelMA/AlgMA) bioprinted scaffolds, and GelMA/AlgMA/hydroxyapatite (GelMA/AlgMA/HAP) bioprinted bone organoids in vitro. A) Schematic representation of the experimental setup. B) Cumulative mineralized tissue quantity (TMV, sample size = 6, ∗statistically significant at p < 0.05, ∗∗p < 0.01, and ∗∗∗p < 0.001). C) Mineralization density within engineered tissue constructs (OMD, n = 6, ∗p < 0.05, ∗∗p < 0.01, and ∗∗∗p < 0.001). D) Macroscopic photograph of 3D-bioprinted matrices after 20-day in vitro culture. E) Gross morphological appearance of biofabricated constructs post 40-day in vitro maintenance. F) Three-dimensional micro-computed tomography visualization of printed scaffolds subsequent to 20-day laboratory cultivation. G) μCT-based volumetric reconstruction of engineered tissue frameworks following 40-day ex vivo incubation [154]. Copyright 2025 Elsevier.

Biomimetic bone tissue engineered scaffolds with a biomimetic architecture can be prepared by combining the biomimetic mineralization process with alginate. Besides, due to its rapid gelling behavior, alginate is widely used in preparing scaffold with intricate architectures [153]. Wang et al. prepared a novel bioink by mixing gelatin methacrylate (GelMA), alginate methacrylate (AlgMA) and hydroxyapatite (HAP). The bioink was then used to print bone organoid scaffolds with intricate shapes using the digital light processing (DLP) based 3D printing method. The GelMA/AlgMA/HAP scaffolds exhibited robust self-mineralization capacities both in vitro and *in vivo*. Scaffolds formed an ectomorphic mineralized structure that was similar to natural bone (Fig. 7) [154]. Neveen et al. have developed perovskite nano-powder in a chemical synthesis procedure and made an ink formulation with 10 % (W/V) alginate, and 10 % (W/W) perovskite. This ink is used to print a scaffold in dimensions of 20 mm × 20 mm × 4.48 mm by using Robota Bio 2 bio-printer, in which the cross-linking agent was a 5 % calcium chloride solution. The generated scaffold, together with MSCs, has considerable potential for clinical application in the treatment of skull defects because it enhances in particular bone mineral density and bone regeneration but has also shown supportive bone-tissue healing and remodeling by facilitating blood vessel formation and inhibiting osteoclast activation [155].

Alginate forms hydrogels via ion cross-linking (e.g., with Ca^2+^ or Sr^2+^), a process that is simple, rapid, and amenable to in situ formation [156,157]. Dual cross-linking strategies, such as combining photo-crosslinking with ion cross-linking, markedly enhance the stability of alginate scaffolds [158]. Lv et al. successfully prepared photo-crosslinkable SAGMA material by reacting sodium alginate (SA) with glycidyl methacrylate (GMA) through chemically route. Then scaffolds were prepared by 4D printing followed by irradiation to UV for photo-crosslinking, then the scaffolds were successively soaked in calcium ion solution and chitosan solution to further crosslink. This three-step crosslinking strategy allows the 4D-printed self-bending SAGMA/nMC scaffold to possess excellent biocompatibility and the ability to support the proliferation and differentiation of osteoblasts, making it a promising method for repairing skull defects [159].

### 4.3 Hyaluronic acid

Hyaluronic acid (HA), a type of glycosaminoglycan, is extensively present in the extracellular matrix and demonstrates outstanding hydrating and lubricating capabilities [160]. It can promote the formation of hydroxyapatite through its interaction with calcium ions, enabling this polysaccharide to induce biomineralization and thereby accelerate bone tissue regeneration [161].

HA plays the vital role in tissue engineering of bones due to its exceptional biocompatibility, degradability and nonimmunogenic [162]. It was then combined with other materials like chitosan, hydroxyapatite, graphene oxide to fabricate the scaffold for bone tissue engineering applications [163]. Kim et al. used 3D printing technology to fabricate Gelatin/HA/HAp composite scaffold. Biological tests also showed that the bioceramalization process significantly enhanced the roughness of the scaffold surface and promoted the deposition of nano-hydroxyapatite, which further promoted cell attachment and growth, indicating that the combination of 3D printing and bioceramalization may offer a promising way for the preparation of the composite scaffolds with higher biocompatibility and superior bone regeneration ability [164]. Afeesh et al. developed an innovative kind of biocompatible osteogenically active scaffold by incorporating graphene oxide (GO), HA, chitosan (CS) and simvastatin (SV) through a freeze-drying and cross-linking method. The results of the SV loaded GO-CS-HA scaffolds showed better properties in terms of porosity, density, swelling behavior, degradation rate, as well as biomineralization ability, compared to other CS and HA based scaffolds. The in vitro experiments indicated that the SV remarkably promoted osteogenesis and biomineralization while having good biocompatibility [165].

HA is an important component of the extracellular matrix and plays an important role in the modulation of many cellular processes, including adhesion, migration, proliferation and differentiation [166]. In literature HA is commonly used as a template for biomineralization to guide mineral such as hydroxyapatite (HAp) on scaffold materials to create a structure that resembles the natural bone tissue [167]. Subramaniam et al. made an alveolar bone re-generation composite material (HAP/CS/HA) which combined HA, HAP and calcium sulfate (CS) with collagenase (Col) as one of its constituents. Hydroxyapatite was synthesized by the coprecipitation technique, then was mixed with the calcium sulfate and hyaluronic acid to form the composites structure, which can be used as a bone replacement material due to its extremely high mechanical performance and biocompatibility to be used in medicine. This material has very high compressive strength. With a strength of 6.69 MPa which is in the comparable range with that of native cancellous bone, 21 min setting time which is compatible with a clinical procedure, and HA induced biomineralization with remarkable new bone formation *in vivo*, CAHA is an excellent candidate for bone regeneration [168].

Hyaluronic acid is frequently utilized for fabrication of hydrogels via both physical or chemical cross-linking approach. It is thus possible to tailor the loading-bearing capability of hydrogel matrix via modulation the cross-linking method, subsequently making it more suitable for osseointegration goals in bone regeneration. Yuki et al. utilized ethylene glycol diglycidyl ether (EGDE) as chemical cross link to individually cross-link hyaluronic acid (HA) and gelatin (Gel). A composite structure of cHA/cGel was finally prepared by mixing cHA and cGel (3:1 vol ratio) through freeze drying technique. Nano-hydroxyapatite and bone morphogenetic protein have also been added to the mixture of cHA/cGel composite which gave the final composite structure. *In vivo* experiments demonstrated that this composite effectively promoted new bone formation and growth in a rat cranial defect and suggested that this material could be a substitute for natural bone [169].

### 4.4 Sulfated polysaccharide

**Fig. 8:**
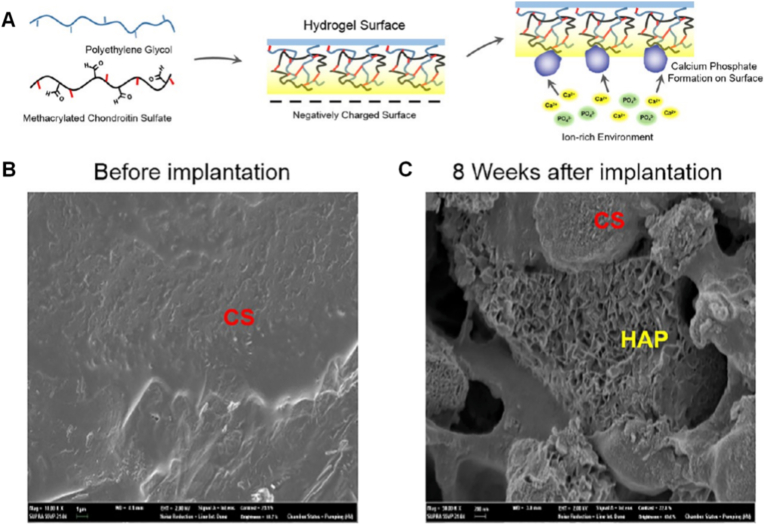
Biomineralization principle and *In vivo* hydroxyapatite forming. A) Schematic diagram of chondroitin sulfate promoting biomineralization B) *In vivo* development of bone mineral through MeCS gel implantation. C) After an 8-week duration, the surface morphology of the hydrogels was analyzed through scanning electron microscopy, revealing the development of hydroxyapatites within the gathered samples [170]. Copyright 2017 American Chemical Society.

Chondroitin sulfate demonstrates a biomimetic approach aligned with biologically controlled mineralization. This sulfated polysaccharide plays a critical role in influencing both the rate and orientation of hydroxyapatite crystallization. Beyond simply promoting mineral deposition, chondroitin sulfate engages in specific molecular interactions with collagen and mineral ions. These interactions allow it to regulate nucleation sites, crystal alignment, and growth kinetics of hydroxyapatite, mirroring the precise control observed in natural biological systems. By adjusting its content and spatial distribution, chondroitin sulfate can be used to direct and fine-tune the biomineralization process with a high degree of specificity [161]. Kim et al. reported methacrylate groups onto chondroitin sulfate (CS) using chemical modification and then co-polymerized with polyethyleneglycol diacrylate (PEGDA) to generate the hydrogel. The PEGDA/CS hydrogels with different CS concentration (0 %, 1 %, 5 %, 10 %) were synthesized as 3D biomineralization scaffold. These hydrogels efficiently chelate Ca^2+^and PO_4_^3−^ ions whereby the anionic character of CS promotes hydroxyapatite crystal growth, thus facilitating a biomimetic mineralization. Interestingly, the 10 % CS hydrogel group showed the greatest in-vitro binding capacity to Ca/P ions as well as strongly inducing an osteogenic differentiation of human tonsil derived mesenchymal stem cells (hTMSCs) (Fig. 8) [170]. Wolfgang et al. studied the effect of adding glycosaminoglycan (GAG) in HA/collagen composite (HA/Col) using a sheep tibia model for their effect on bone remodeling and bone implant interaction, finding that addition of CS greatly improved bone tissue repair and regeneration; with proven effectiveness in a large animal model [171].

Sulfated polysaccharide scaffolds efficiently bind and release key growth factors (e.g., BMP-2, TGF-β), prolonging their biological activity. Additionally, by mimicking the physical and molecular properties of the extracellular matrix, these scaffolds promote stem cell attachment, proliferation, and differentiation, thereby enhancing tissue repair potential [172].

κ-carrageenan (κ-CG) is an anionic, sulfated polysaccharide which has a surface that is predominantly acidic in nature containing functional groups such as sulfate (-SO_4_^2-^) and hydroxyl (-OH). These functional groups could interact with calcium (Ca^2+^) ions and phosphate ions (PO_4_^3−^), offering a template for biomineralization processes. Nowsheen et al., fabricated a series of biomolecular scaffolds based on polyhydroxybutyric acid (PHB) and valerate polyhydroxybutyrate (PHBV) materials via electrospinning process with the addition of different amount of κ-CG. Experimental observations demonstrated a positive correlation between κ-CG concentration gradients and augmented surface hydration capacity across three-dimensional biomaterial frameworks. Additionally, the crystal morphology shifted from micrometer-level (7 μm) to nanometer-level (around 800 nm). Furthermore, both cell proliferation and mineralization ability were substantially enhanced. The integration of PHBV and κ-CG markedly enhances the biological performance and osteogenic differentiation potential of the scaffold [173].

Sulfated polysaccharide is a promising material in the application of scaffold for bone tissue engineering, because an ideal scaffold for tissue engineering should possess adequate mechanical properties, precise dimensions, correct porosity [174]. Sulfated polysaccharide scaffolds can effectively mimic the properties of natural tissues such as cell binding epitopes or mechanical characteristics. They also offer specific places where the mineral of bones can be built up [172,174]. Ulvan is a sulfated polysaccharide extracted from green algae. Stefanos et al. developed a hybrid scaffold (UP scaffold) using a polycaprolactone - based biomaterial. This was accomplished by dissolving ulvan in water and then blending it with a PCL solution to create an emulsion. The porous structure of the PCL scaffolds, featuring pore sizes between 50 and 300 μm and high porosity (79 %–82 %), meets the criteria for bone tissue engineering. Additionally, the addition of ulvan considerably improved the water absorption capability of the scaffolds and enhanced the osteogenic induction potential of the PCL scaffolds [175].

Sulfated polysaccharides have also been found to play a role alongside other biomaterials in improving other functionalities. For example, sulfated polysaccharides (SPS) have been used combined with bioceramic material such as hydroxyapatite (HA) to mimic the organic and mineral components of natural bone tissue and accelerate bone regeneration [151]. Hu et al. developed a composite scaffold with nano-hydroxyapatite, chitosan, chondroitin sulfate and hyaluronic acid (nHAP/CS/CSA/HA) with a hierarchical micro/nan architecture using biomimetic mineralization and freeze-drying technologies. Their structure showed a uniform porous morphology, their pore sizes ranging from 45 to 156 μm and its porosity of 93.78 %. In addition, they exhibited also very good mechanical properties, including doubled compressive stress and modulus of elasticity. In vitro mineralization experiments showed growth of osteoid-like mineral crystal deposits onto the scaffold surface. In addition, the biocompatibility tests demonstrated that the osteoblasts showed good proliferation and differentiation abilities on the scaffold and a notable increase in the activity of the alkaline phosphatase leading to the good osteogenic induction [176].

### 4.5 Cellulose

Cellulose and its derivative compounds are of great value for biomineralization [177], of advantages such as a variety of source materials, good biocompatibility and a tunable physicochemical characteristics [178]. As a natural polysaccharide, cellulose is derived from multiple sources (e.g., plants, bacteria, algae). Cellulose nanocrystals (CNCs) and cellulose nanofibers (CNFs) contain surface hydroxyl and carboxyl groups that readily interact with Ca^2+^, facilitating hydroxyapatite formation [179]. Cellulose is one of the abundant biomaterials that are widely utilized for bone tissue engineering applications due to its usage as a three-dimensional scaffold where bone cells are embedded in a three-dimension microenvironment. This environment facilitates cell growth, enhances cell adhesion proliferation and differentiation that will lead to bone tissue regeneration [180].

As cellulose can be directly converted into different scaffold architectures, such as nanofiber scaffold [180], which are applicable for filling bone defects and guiding bone tissue regeneration [181]. Nourany's research group developed a 3D scaffold with the double porosity, it reveals higher capability of promoting growth kinetic and enhancing osteogenic differentiation potential of hBMSCs. They accomplished this by compounding poly(ε-caprolactone) (PCL) and polylactic acid (PLA) in a proportion of 70:30, incorporating three types of PCL-PEGx- PCL triblock copolymers and incorporating cellulose nanocrystals. It showed that the addition of CNCs drastically enhanced the biomineralization capacity of the scaffolds, strongly promoted the osteogenic differentiation of hBMSCs and clearly increased the levels of ALP activity and calcium deposition. Furthermore, the gene expression analysis showed that CNC-enhanced scaffolds significantly elevated the expression of bone related genes such as BGLAP (osteocalcin) and BMP4 [182]. Hamouda et al. synthesized composite nanofiber scaffolds through electrospinning of cellulose acetate (CA) with low concentration of iron acetate (0.5 wt%) for the application in the bone tissue engineering. This type of scaffold with iron nanoparticles can be successfully fabricated by simply introducing iron acetate on a 17 wt% CA solution followed by electrospinning, and in high direct current (DC) voltage. The results demonstrated the iron acetate reduced the mean diameter of fibers from 395 nm to 266 nm which results in a tightly packed fiber structure similar to natural ECM. Additionally, the scaffold exhibited good ability to support the adhesion and proliferation of human fetal osteoblasts (hFOB) as a highly osteogenic scaffold [183].

**Fig. 9:**
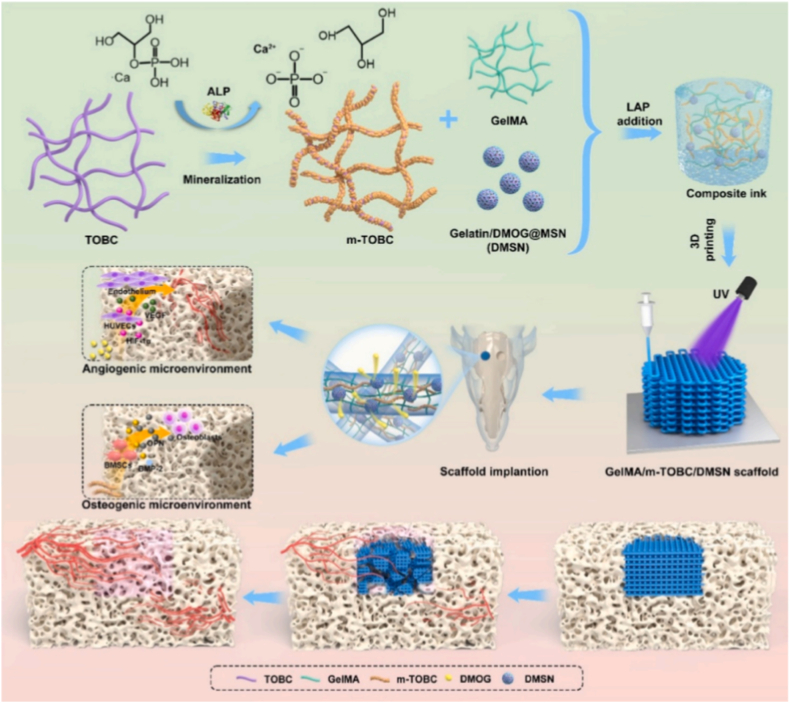
Schematic representation of the development of a bioactive composite GelMA/m-TOBC/DMSN hydrogel designed for cranial repair. The GelMA/m-TOBC/DMSN system is capable of reshaping the osteogenic and angiogenic microenvironment by continuously releasing DMOG and biomineral ions throughout the bone defect repair process [185]. Copyright 2025 Elsevier.

Bacterial cellulose (BC) has attracted substantial attention in the domain of bone tissue engineering. This is due to its distinctive nanofiber structure, remarkable mechanical properties, superior water retention capacity, and excellent biocompatibility [180]. Ana et al. utilized bacterial nanocellulose (BNC) as the basic material to fabricate a three - dimensional scaffold with a microporous structure. This was accomplished by mineralizing the BNC in calcium and phosphate solutions via an alternating immersion process. With the increase in the number of mineralization cycles, both the size and density of calcium phosphate crystals on the surface of the scaffold increased remarkably. The scaffolds exhibited excellent cell adhesion abilities, maintaining a cell viability as high as 95 %. Moreover, they successfully promoted the osteogenic differentiation of human bone marrow mesenchymal stem cells [184]. Liu et al. succeeded in coating hydroxyapatite on top of bacterial cellulose nano fibers using 2,2,6,6-tetramethylpiperidin-1-oxide (TEMPO)-mediated oxidation and enzymatic mineralization methods, resulting in mineralized TEMPO-oxidized bacterial cellulose nano fibers (m-TOBC). Subsequently, these m-TOBC nanofibers were blended with dimethyloxalylglycine (DMOG) loaded mesoporous silicon nanoparticles (DMSN) in GelMA hydrogel. Using the 3D bioprinting method, we fabricated the bioinspired hydrogel scaffolds with well-defined structures and functionalities. Our composite hydrogel was found to be highly printable, biocompatibility, mechanical strength. The results from the in vitro study demonstrated that the hydrogel promoted significantly the formation of the mineralization matrix, as well as the activity of alkaline phosphatase by osteoblasts; furthermore, *in vivo* studies using a rat calvarial defect model showed that the hydrogel was effective for promoting bone regeneration (Fig. 9) [185].

### 4.6 Other common polysaccharides

Xanthan gum is a polysaccharide synthesized through bacterial fermentation, which exhibits nontoxicity to human cells and undergoes gradual degradation in the body [186]. Because of its outstanding mechanical strength and stability, xanthan gum has found widespread application in bone tissue engineering, where it satisfies the rigorous mechanical requirements necessary for such applications. Liu et al. created an injectable, mineralizable, and drug-carrying hydrogel using gelatin methacryloylation (GM) and altered xanthan gum (CMXG/SXG). Following mineralization, the CMXG/SXG-GM hydrogel exhibited high crystallinity of hydroxyapatite, excellent injectability, and biocompatibility [113].

Sea urchin polysaccharide (SUP) is a polysaccharide extracted from the sea urchin with good biocompatibility, degradability, and biological activity. As its conformation is similar to the human extracellular matrix, it can promote cell adhesion, proliferation and differentiation, making it potential biomedical application for bone tissue-engineering [187]. To further enhance the capability of a scaffold to induce BMP-2 release and enable them to help induce an injury's bone healing, Sun et al. used BMP-2 to form controlled released system by integrating BMP-2 and non-sulfate polysaccharide (SUP) into calcium phosphate cement (CPC) scaffolds; they may allow a continuous induction of BMP-2/Smads and Runx-2 signal pathways during this process. Their experiments showed that the CPC-BMP2-SUP scaffold can continuously release BMP-2 for 21 d. It induced high enhancement in alkaline phosphatase enzymatic activity and elevated osteogenesis-related gene transcription. And the *in vivo* trial also corroborated that the scaffold effectively reduced the bone defect area, enhanced bone surface features, and increased bone volume [188].

Noggin is a natural polysaccharide containing high content of the reactive functional groups such as hydroxyl and carboxyl groups, which can be associated with other biomolecules to form stable complex. Additionally, Noggin also has been proved to repair the skeletal tissue by regulating essential biological mechanisms such as cell proliferation, differentiations, and migration [189]. Zhou et al. created a ternary composite scaffold for bone tissue regeneration, utilizing gellan gum (GG) as the primary material. This was achieved by integrating different concentrations (1, 3, and 5 wt%) of Ti_3_C_2_T_X_ MXene nanosheets along with garlic extract (GA) into the GG framework. The experimental results revealed that the scaffold exhibited a smooth and uniform surface, optimized microstructure, an increased amorphous region (15 %), enhanced hydrophilicity (contact angle of 27.5°), a suitable Young's modulus (13.43 MPa), as well as remarkable osteogenic adhesion and antibacterial properties [190].

The carboxymethyl tamarensis polysaccharide, a natural polysaccharide, it has the characteristic of good biocompatibility, degradability, and the environment is suitable for the growth of bone cell [151]. Singh et al. used carboxymethyl tamarectin (CMT) as matrix to prepare a composite material with nano-hydroxyapatite particles embedded in a polymer network by polymerization reaction initiated by 2-hydroxyethyl methacrylate (HEMA) and benzoyl peroxide. The resultant biomaterial features a highly porous architecture, with pore dimensions varying between 50 and 140 μm, a porosity level of 94.73 %, outstanding biocompatibility, and the capacity to significantly enhance the in vitro mineralization of osteoblast [191].

### 4.7 Comparative insights on representative polysaccharides in bone-tissue engineering

A side-by-side comparison of the most frequently used types of polysaccharides - chitosan, alginate, hyaluronic acid, chondroitin sulfate, and nano- or bacterial cellulose-evidently reveals the tradeoff between "biological activity" and "mechanical properties". Chitosan and HA exhibit remarkable biological activities. The positively charged surface of chitosan can accumulate phosphate and calcium ions via electrostatic interaction [130], and facilitate osteoblast adhesion through integrin mediation [131]. HA, on the other hand, speeds up cell migration and angiogenesis through the CD44 signaling pathway [162]. However, the compression modulus of pure chitosan hydrogels is less than 2 kPa [145], and that of HA hydrogels is less than 1 kPa [164]. Before being applied to the force-bearing site, their compression modulus needs to be improved. This can be achieved through methods such as adding nano-hydroxyapatite [145], bioactive glass [134], or using methacrylate crosslinking [141].

Alginate presents complementary characteristics. Its ion gelation triggered by Ca^2+^ [149]and non - Newtonian rheological properties render it a favored option for injectable or 3D printed structures [154]. However, the same unstable ion cross - linking leads to rapid swelling and an abrupt loss of strength at physiological ionic strength [158]. In contrast, cellulose nanofibers and bacterial cellulose are on the opposite side. Their crystalline frameworks are capable of providing a tensile modulus exceeding 10 GPa [182]. Nevertheless, their bioinert surfaces and *in vivo* enzymatic inertness necessitate oxidation or peptide grafting to initiate an osteogenic response [185]. Chondroitin sulfate holds a distinct position: its high - density sulfate group can directionally promote the nucleation of hydroxyapatite within the collagen gap [170].

Degradation kinetics serve to further distinguish these materials. Alginate can be dissolved via ion exchange within a few weeks [149]. This time frame aligns with the period of early osteogenic induction of encapsulated stem cells [155]. Bacterial cellulose can persist for several years, making it appropriate for large segmental defects that demand long-term mechanical support. HA can be degraded by hyaluronidase. By adjusting the density of methacrylate, its half-life can be prolonged from hours to months [169]. This offers a temporal control that is not achievable with the chitosan acid hydrolysis mechanism, unless extra cross-linking agents are added [141].

Regarding the mechanism of action, these polysaccharides do not compete with one another but are complementary. When positively charged chitosan and negatively charged chondroitin sulfate or alginate are assembled in layers, Ca^2+^ enrichment, crystal orientation, and the display of osteoblast integrin binding sites can be accomplished simultaneously. This integrates ion chelation, template orientation, and cell recruitment within the same hierarchical interface [176].

**Fig. 10:**
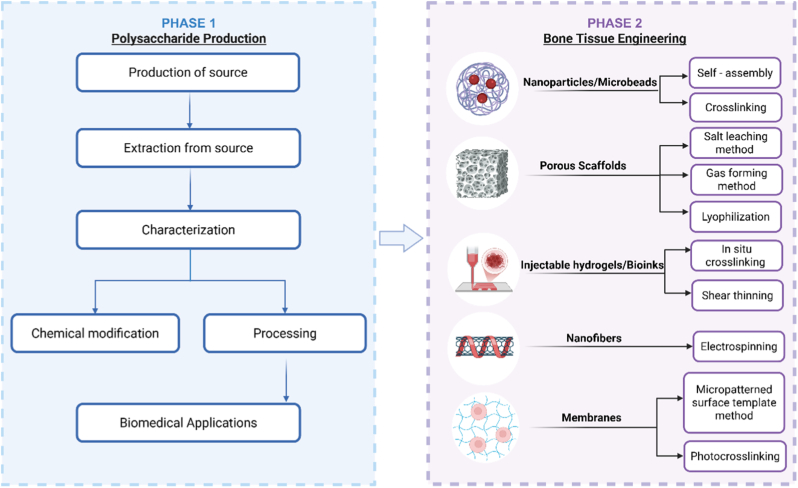
General procedures for the production and processing of polysaccharides. Polysaccharides can be used in various forms for bone tissue engineering (BTE) applications. These forms include nanoparticles, scaffolds, bioinks, nanofibers and membranes. Created with biorender.

Concerning clinical transformation, polysaccharides obtained from plants or microorganisms, such as cellulose, alginic acid, and bacterial cellulose, carry a lower risk of pathogens, and the downstream large-scale processing is well-established [182,185]. Conversely, animal-derived chondroitin sulfate has to confront ethical and regulatory obstacles [171]. Further investigations should focus on designing dual cross-linked alginate or hyaluronic acid network. This could address the need for longer term mechanical requirements without sacrificing rapid injectability [159,169], as well as the use of surface functionalized cellulose nanofibers that can add an additional reinforcing phase with high modulus and bioactivity [185]or recombinant or chemically enzymatically produced analogs of chondroitin sulfate. This method will break through the bottleneck of animal-derived raw material resources and maintain its original purpose, that is template induced mineralization [175]. We should use the inherent property of polysaccharides to improve its own functional effect with a more reasonable way and strategy (Fig. 10) [192].

An evaluation of the primary polysaccharides used in bone tissue engineering reveals a fundamental trade-off between their structural strength and biological activity. To facilitate rapid access to key attributes and address current informational deficiencies, the table below consolidates vital information—including mineralization mechanisms, principal advantages, significant drawbacks, degradation behavior, and clinical translation prospects—all directly extracted from the existing literature (Table 3).

**Table 3:** Comparative Overview of polysaccharide-based materials in bone tissue engineering.

Polysaccharide	Mineralization Mechanism	Mechanical Properties	Limitations	Clinical Translation Potential
Chitosan	Electrostatic adsorption of Ca^2+^/PO_4_^3−^, providing nucleation sites	Pure hydrogel compressive modulus <2 kPa, modifiable for enhancement	Insufficient mechanical strength; requires crosslinking for degradation regulation	Wide source, low toxicity, mature large-scale processing
Alginate	Ca^2+^-crosslinked gel formation, mimicking ECM microenvironment	Unstable ion crosslinking, prone to swelling and strength loss	Rapid degradation; limited mechanical strength of pure materials	Seaweed-derived, low risk, suitable for injectable/3D-printed products
Hyaluronic Acid (HA)	Binds Ca^2+^ to promote hydroxyapatite formation; CD44 pathway accelerates angiogenesis	Extremely low mechanical strength (<1 kPa)	Poor strength, rapid degradation; requires composite modification	Wide source, chemically modifiable, suitable for bone-soft tissue integration repair
Chondroitin Sulfate (CS)	Sulfate groups directionally induce hydroxyapatite nucleation in collagen gaps	No inherent mechanical advantages; dependent on composite matrix	Animal-derived, ethical/batch variability issues	Unique mineralization regulation; needs recombinant/synthetic analogs to overcome raw material bottlenecks
Nano/Bacterial Cellulose	Surface hydroxyl/carboxyl groups bind Ca^2+^, providing mineralization templates	Bioinert surface; enzymatic inertness *in vivo*; requires oxidation/peptide grafting	Bioinert surface; requires oxidation/peptide grafting modification	Wide source, low risk, applicable for large bone defects; surface functionalization required

### 4.8 Comparative analysis: roles of polysaccharides and other natural macromolecules in bone tissue engineering

Polysaccharides have great promise as mediators of biomineralization during bone regeneration but the extent to which they succeed should be compared with that of other major natural macromolecules such as collagen or gelatin. The recent progress on biomaterial design has shed light onto the individual benefits and drawbacks of different material classes, thus helping in the choice of adequate material to be used as a bone tissue engineering application. Since collagen is the main organic constituent of the native bone matrix and inherently bioactive by virtue of its integrin-binding motifs (e.g., RGD sequences), that stimulate cell adhesion and osteogenic differentiation [154]. However, pure collagen scaffolds exhibit fast degradation rates and typically do not possess sufficient mechanical strength to be used in a load bearing application. Chemical modification strategies such as methacrylation, gelatin methacryloyl (GelMA), which greatly enhances mechanical strength and robustness in collagen-based scaffolds. For example, the group of Wang et al. reported that a GelMA/AlgMA/HAP composite bioink could be 3D-printed into large-scale bone-like organoids capable of self-mineralization, to achieve a compressive modulus of 0.414 GPa, which is comparable with that of cancellous bone, after 40 days in culture [154]. The incorporation of hydroxyapatite into GelMA matrices increased mineral content to 40.67 % and enhanced the expression of osteogenic markers (RUNX2, OCN, OPN). Likewise, it is possible to chemically functionalize gelatinbased systems. For example, in GelMA hydrogels reinforced with peptides fabricated by Huang and coworkers, the addition of a nucleating domain peptide (HGRGEAFDY), increased in situ mineralization saturation from 29.4 % to 56.3 %, and compressive strength increased from 9.06 MPa to 15.79 MPa [193]. This strategy took advantage of the synergy effect between the peptides as nucleation site and the gellan matrix in order to increase both mineralization efficiency and mechanical properties at once.

Unlike the proteins-derived materials mentioned above, polysaccharides have better processability and less immunogenicity. By introducing "freezing-salting-mineralization" strategy, Xu et al. constructed modulated mineralized hydrogels with nine kinds of biomedical polymers, including chitosan, sodium alginate and cellulose derivatives [194]. This strategy was proven to allow efficient biomineralization using polysaccharides and no complex chemical modifications were needed, producing collagen/HA hydrogels having a compressive modulus of 36.79 kPa, and a mineral content of 43.41 wt%. Enzymatic mineralization is also a possible comparison point. Ling et al. combined the use of anionic enhancement with enzymatic mineralization in GelMA-AlgMA composite hydrogels, by using polyaspartic acid in order to increase the number of nucleation sites [195]. This strategy increased the in situ mineralization saturation level by about 5 wt%, and an *in vivo* cranial defect model showed a much greater amount of bone volume fraction on the pAsp-enhanced scaffold than that on control ones (25.5 ± 1.2 % versus 20.6 ± 1.3 %) after 4 weeks [195]. Remarkably, this work highlighted how polysaccharidic elements (e.g., alginate) can add value in a synergy way within composite system together with protein-derived element like gelatine.

Taken together, the results demonstrate that because of its tuneable rheological characteristics and fast gelation dynamics, polysaccharide can be better suited for the purpose of 3D bioprinting. Bioinks based on alginate possess a higher structural integrity during printing than those from collagen alone. Furthermore, polysaccharides are abundant with hydroxyl and carboxyl groups that can be chemical modified forallowing for tailored degradation behaviour and mineral nucleation whilst retaining the advantages of collagen and gelatin with regards to their intrinsic bioactivity, which are due to the fact that they contain natural cell adhesive moieties, which promote adhesion and growth of bone forming cells (osteoblasts). In contrast with polymeric scaffolds based on polysaccharides that usually need further surface modification for cell adhesion, native binding sites on collagen-based scaffolds generally promote faster cell seeding and tissue growth. As far as the mechanism of mineralization is concerned, it seems that polysaccarides mainly use an electrostatic interaction and a templating effect in directing HA precipitation, whereas collagen-based scaffolds can support a more physiological type of mineralization due to the fibrous structure, which is similar to natural bone extracellular matrix. Nevertheless, chemically modified polysaccharides provide more flexible opportunities of tuning the environment for a particular regeneration need. The synthesis of existing literature indicates the need to choose materials based on their intended use. For structural purposes, for applications that require balanced mechanical properties, cross-linked polysaccharide-based materials and/or polysaccharide-gelatin blends might be more suitable as they are able to offer better mechanical properties than both individual components. On the other hand, for those applications where the emphasis is on a fast incorporation into cells and early bone-forming activity, collagen- or gelatin-based materials would perhaps be preferred. Finally, it can be seen that there is an emerging interest for more hybrid approaches combining advantages from different types of materials such as the one we reported recently with sodium alginate and GelMA [154,195]. Although polysaccharides, collagen and gelatin have their own advantages for use as a single component in bone tissue engineering, multifunctional multi-component systems combining the processing flexibility and controllability of polysaccharides and the natural bioactivities of proteins are leading to novel biomineralized scaffolds towards better clinical applications.

## 5 Conclusions

Herein, we revisit biomineralization paradigm highlighting the critical role of polysaccharides in facilitating biomineralization. Through this review, we explore the use of polysaccharide-derived materials in bone tissue engineering by explaining how polysaccharides influence and control biomineralization. This analysis will contribute to a more comprehensive understanding of polysaccharide-based materials and their pivotal significance in biomineralization.

Polysaccharides have enormous potential for controlling the biomineralization process or as a means of delivering scaffolds to facilitate bone tissue engineering; however, there are key issues that need addressing: including low mechanical strength and structural instability, lack of precise spatiotemporal control over the mineralization, and biological inertness. The majority of naturally occurring polysaccharide hydrogels possess a compressive modulus <2 kPa, which does not satisfy the mechanical needs of weight bearing bone defects. In addition, the calcium cross-linked alginate or hyaluronic acid gels easily swell and degrade in body fluid, resulting in early collapse of the scaffolds. Although the bacterial cellulose and some plant polysaccharide have good mechanical performance, their biological inertness, requiring a surface modification for enhancing the cell adhesion and osteogenic induction. In addition to this, animal-based chondroitin sulfate and derivatives are limited due to ethical concerns, risk of pathogens, and inter-lot variability which hampers the clinical translation.

The combination of 4D printing with polysaccharide-related materials can effectively overcome the problem of insufficient mechanical strength in bone tissue engineering. The biomimetic porous structure constructed through 4D printing can further optimize the mechanical conductivity and make up for the mechanical strength shortcomings of pure gelatin materials [196]. The material formed by using chitosan as the base and combining with nanocellulose to form a three-dimensional interwoven network not only retains the natural advantages of polysaccharides, such as low toxicity and high biocompatibility, but also takes advantage of the excellent fiber reinforcement effect of nanocellulose. By adapting to the DIW type 4D printing technology, it can precisely construct biomimetic porous structures of bone trabeculae, and after forming, the structural integrity is good and the surface charge is stable, effectively avoiding the defect of easy fracture and insufficient mechanical support of pure polysaccharide materials. The interwoven effect of nanocellulose fibers can significantly enhance the tensile strength of the material, and combined with the biomimetic structure of replicating bone trabeculae by 4D printing, it further optimizes the mechanical conduction path, making the scaffold mechanical properties match those of natural bone tissue and meeting the mechanical support requirements for bone defect repair, thereby overcoming the limitation of weak mechanical strength of pure polysaccharide materials [197].

Artificial Intelligence (AI) can empower polysaccharide-based materials through three dimensions: precise design, process optimization, and real-time control [198]. This approach provides a robust strategy for addressing the challenges associated with controlling both the timing and spatial organization in bone tissue engineering. By processing information such as the extent of polysaccharide material modification and the concentration of cross-linking agents, artificial intelligence can precisely forecast the scaffold's degradation profile and the release dynamics of growth factors. This facilitates a temporal alignment between the material's functional properties and the distinct phases of bone repair. Furthermore, utilizing 3D scan data of bone defects, AI optimizes key parameters in digital light processing (DLP) printing, including light intensity and layer thickness. This optimization enables the pore architecture and mechanical properties of the polysaccharide scaffold to exhibit a gradient distribution in space, thereby mimicking the inherent heterogeneity of natural bone tissue. The system can also incorporate data from biosensors to track the regeneration process in real-time, allowing for dynamic adjustments to the material's responsiveness and ensuring synchronized temporal control over the bone healing progression [199].

The patient-derived mesenchymal stem cell cultures could be cocultivated on the 4D printed bioinspired scaffold and thus create a model of a callus organoid, after which an AI microfluidic chip could be used to mimic the microenvironment of the defect, and through a highthroughput screen on the pro-angiogenic osteogenesis coupling strategy, the likelihood for failure in preclinical trials could be reduced [200].

In conclusion, polysaccharide-based bio-mineralization is a superior concept of bone tissue engineering beyond just the structural and/or composition standpoint, but because of its embodiment of a living, bioresponsive and programmable material philosophy. In contrast to conventional implants and inert scaffolds, polysaccharide platforms permit dynamic control over mineralization, vascularization, and cellular behaviours via their intrinsic chemical versatility, environmental sensitivity and biological dialog capability. In the future, we believe that the combination with 4D printing technology, AI-assisted design and personalized organoids would make the "high-end" of polysaccharide-based systems even more outstanding, shifting them from structural surrogates to smart, responsive and tailored regenerative entities. Therefore the "advanced" quality is not just about what these materials are, it is also about what they can become scaffolds which learn, adapt and evolve with the healing process.

As detailed in the comparative analysis (Section 4.8), the strategic selection and combination of biomaterials are of critical importance. Polysaccharides, collagen, and gelatin all offer unique benefits where the polysaccharides show improved processability and tunable mineralization directionality in comparison to, collagen and gelatin have stronger inherent bioactivities that support cell adhesion. The emergent trend today, however, is to move toward hybrid systems capable of combining synergistically the merits of these materials. For instance, the coupling between sodium alginate and GelMA allows for creating scaffolds which achieve an equilibrium in terms of mechanical characteristics, effective production facilities, and greater biocompatibility. Relative to use of one class of polymers, this multimaterial strategy is, therefore, an even better prospect of future evolution.

## CRediT authorship contribution statement

**Huxin Tang:** Investigation, Writing – original draft, Writing – review & editing. **Mingyang Hu:** Investigation, Writing – original draft, Writing – review & editing. **Xinying Huang:** Investigation, Writing – original draft, Writing – review & editing. **Jianan Chen:** Writing – review & editing. **Yesheng Jin:** Writing – review & editing. **Shuo Chen:** Funding acquisition, Writing – review & editing. **Ke Li:** Funding acquisition, Writing – review & editing. **Yong Xu:** Funding acquisition, Writing – review & editing.

## Declaration of competing interest

The authors declare that they have no known competing financial interests or personal relationships that could have appeared to influence the work reported in this paper.

## Data Availability

No data was used for the research described in the article.

## References

[1] Doyle M.E., Dalgarno K., Masoero E., Ferreira A.M. (2022). Advances in biomimetic collagen mineralisation and future approaches t o bone tissue engineering. Biopolymers.

[2] Hao S., Wang M., Yin Z., Jing Y., Bai L., Su J. (2023). Microenvironment-targeted strategy steers advanced bone regeneration, materials today. Bio.

[3] Stark H.H., Rickard T.A., Zemel N.P., Ashworth C.R. (1988). Treatment of ununited fractures of the scaphoid by iliac bone grafts and kirschner-wire fixation. J Bone Joint Surg Am.

[4] Stavropoulos A., Marcantonio C.C., de Oliveira V.X.R., Marcantonio É., de Oliveira G. (2023). Fresh-frozen allogeneic bone blocks grafts for alveolar ridge augmentation: biological and clinical aspects. Periodontol. 2000.

[5] Wang W., Liang X., Zheng K., Ge G., Chen X., Xu Y., Bai J., Pan G., Geng D. (2022). Horizon of exosome-mediated bone tissue regeneration: the all-rounder role in biomaterial engineering. Mater. Today Bio.

[6] Liu H., Chen H., Han Q., Sun B., Liu Y., Zhang A., Fan D., Xia P., Wang J. (2023). Recent advancement in vascularized tissue-engineered bone based on materials design and modification. Mater. Today Bio.

[7] G.L. Koons, M. Diba, A.G. Mikos,Materials design for bone-tissue engineering, Nat. Rev. Mater. 5(8) 584-603. 10.1038/s41578-020-0204-2.

[8] Q. Zhu, H. Jiao, K. Zhao, Y. Tang, X. Zhao, P. Wang, Preparation and mechanical properties of biomimetic mineralization col lagen for dopamine-polyacrylamide-induced small intestinal submucosa,Mater. Lett. 361 136134. 10.1016/j.matlet.2024.136134.

[9] Li Q., Liu W., Hou W., Wu X., Wei W., Liu J., Hu Y., Dai H. (2023). Micropatterned photothermal double-layer periosteum with angiogenesis-neurogenesis coupling effect for bone regeneration. Mater. Today Bio.

[10] Sarwar T., Raza Z.A., Nazeer M.A., Khan A. (2024). Synthesis of aminolyzed gelatin-mediated chitosan as pH-responsive drug-carrying porous scaffolds. Int. J. Biol. Macromol..

[11] Radulescu D.E., Neacsu I.A., Grumezescu A.M., Andronescu E. (2022). Novel trends into the development of natural hydroxyapatite-based polymeric composites for bone tissue engineering. Polymers.

[12] Amorim S., Reis C.A., Reis R.L., Pires R.A. (2021). Extracellular Matrix mimics using Hyaluronan-Based biomaterials. Trends Biotechnol..

[13] Chen Y., Feng Y., Deveaux J.G., Masoud M.A., Chandra F.S., Chen H., Zhang D., Feng L. (2019). Biomineralization forming process and bio-inspired nanomaterials for biomedical application: a review. Minerals.

[14] Debnath A., Mitra S., Ghosh S., Sen R. (2024). Understanding microbial biomineralization at the molecular level: recent advances. World J. Microbiol. Biotechnol..

[15] Guido A., Sposato M., Palladino G., Vescogni A., Miriello D. (2022). Biomineralization of primary carbonate cements: a new biosignature in the fossil record from the Anisian of Southern Italy. Lethaia.

[16] Görgen S., Benzerara K., Skouri-Panet F., Gugger M., Chauvat F., Cassier-Chauvat C. (2020). The diversity of molecular mechanisms of carbonate biomineralization by bacteria. Discov. Mater..

[17] Cuéllar-Cruz M. (2017). Synthesis of inorganic and organic crystals mediated by proteins in different biological organisms. A mechanism of biomineralization conserved throughout evolution in all living species. Prog. Cryst. Growth Char. Mater..

[18] Falini G., Fermani S., Goffredo S. (2015). Coral biomineralization: a focus on intra-skeletal organic matrix and calcification. Semin. Cell Dev. Biol..

[19] Yao S., Jin B., Liu Z., Shao C., Zhao R., Wang X., Tang R. (2017). Biomineralization: from material tactics to biological strategy. Adv. Mater..

[20] Qin D., Leichner C., Delakis M., Fornoni M., Luo S., Yang F., Wang W., Banbury C., Ye C., Akin B., Aggarwal V., Zhu T., Moro D., Howard A. (2025).

[21] Zhang X., Fussenegger M. (2024). Structural materials meet synthetic biology in biomedical applications. Mater. Today.

[22] Maré A., D'Haese P.C., Verhulst A. (2020). The role of sclerostin in bone and ectopic calcification. Int. J. Mol. Sci..

[23] Onnis C., Virmani R., Kawai K., Nardi V., Lerman A., Cademartiri F., Scicolone R., Boi A., Congiu T., Faa G., Libby P., Saba L. (2024). Coronary artery calcification: current concepts and clinical implications. Circulation.

[24] Fãgãrãşan A., Gozar L., Ghiragosian S.R., Murariu M., Pop M., Crauciuc A., Miclea D., Şuteu C.C. (2022). Severe early-onset manifestations of generalized arterial calcification of infancy (mimicking severe coarctation of the aorta) with ABCC6 gene variant - case report and literature review. Front. Cardiovasc. Med..

[25] Kawai K., Sato Y., Kawakami R., Sakamoto A., Cornelissen A., Mori M., Ghosh S., Kutys R., Virmani R., Finn A.V. (2022). Generalized arterial calcification of infancy (GACI): optimizing care with a multidisciplinary approach. J. Multidiscip. Healthc..

[26] Nollet L., Van Gils M., Willaert A., Coucke P.J., Vanakker O.M. (2022). Minocycline Attenuates excessive DNA damage response and reduces ectopic calcification in pseudoxanthoma Elasticum. J. Invest. Dermatol..

[27] Sherwood J. (2019). Osteoarthritis year in review 2018: biology. Osteoarthr. Cartil..

[28] Hahn D., Hodson E.M., Craig J.C. (2015). Interventions for metabolic bone disease in children with chronic kidney disease. Cochrane Database Syst. Rev..

[29] Zimmerman K., Liu X., von Kroge S., Stabach P., Lester E.R., Chu E.Y., Srivastava S., Somerman M.J., Tommasini S.M., Busse B., Schinke T., Carpenter T.O., Oheim R., Braddock D.T. (2022). Catalysis-Independent ENPP1 protein signaling regulates Mammalian bone mass. J. Bone Miner. Res..

[30] Zhang K., Tang C.S., Jiang N.J., Pan X.H., Liu B., Wang Y.J., Shi B. (2023). Microbial-induced carbonate precipitation (MICP) technology: a review on the fundamentals and engineering applications. Environ. Earth Sci..

[31] Rajasekar A., Wilkinson S., Moy C.K.S. (2021). MICP as a potential sustainable technique to treat or entrap contaminants in the natural environment: a review. Environ. Sci. Ecotechnol..

[32] Lin H., Zhou M., Li B., Dong Y. (2023). Mechanisms, application advances and future perspectives of microbial-induced heavy metal precipitation: a review. Int. Biodeterior. Biodegrad..

[33] Dong H., Huang L., Zhao L., Zeng Q., Liu X., Sheng Y., Shi L., Wu G., Jiang H., Li F., Zhang L., Guo D., Li G., Hou W., Chen H. (2022). A critical review of mineral-microbe interaction and co-evolution: mechanisms and applications. Natl. Sci. Rev..

[34] Bazylinski D.A., Frankel R.B. (2004). Magnetosome formation in prokaryotes. Nat. Rev. Microbiol..

[35] Zhang Y., Ma S., Nie J., Liu Z., Chen F., Li A., Pei D. (2024). Journey of mineral precursors in bone mineralization: evolution and inspiration for biomimetic design. Small.

[36] Xiao Y., He X., Wu W., Stuedlein A.W., Evans T.M., Chu J., Liu H., van Paassen L.A., Wu H. (2021). Kinetic biomineralization through microfluidic chip tests. Acta Geotech..

[37] Delgado G., Delgado R., Párraga J., Rivadeneyra M.A., Aranda V. (2008). Precipitation of carbonates and phosphates by bacteria in extract solutions from a semi-arid saline soil. Influence of Ca2+and Mg2+Concentrations and Mg2+/Ca2+Molar Ratio in Biomineralization, Geomicrobiology Journal.

[38] Li S., Huang J., Liu C., Liu Y., Zheng G., Xie L., Zhang R. (2016). Interactive effects of seawater acidification and elevated temperature on the transcriptome and biomineralization in the pearl oyster Pinctada fucata. Environ. Sci. Technol..

[39] Guo J., Yao H., Chang L., Zhu W., Zhang Y., Li X., Yang B., Dai B., Chen X., Lei L., Chen Z., Li Y., Zheng L., Liu W., Tong W., Su Y., Qin L., Xu J. (2025). Magnesium nanocomposite hydrogel reverses the pathologies to enhance mandible regeneration. Adv. Mater..

[40] Morrison K.D., Zavarin M., Kersting A.B., Begg J.D., Mason H.E., Balboni E., Jiao Y. (2021). Influence of uranium concentration and pH on U-Phosphate biomineralization by caulobacter OR37. Environ. Sci. Technol..

[41] Chen M., Li Y., Zhang M., Ge S., Feng T., Chen R., Shen J., Li R., Wang Z., Xie Y., Wang D., Liu J., Lin Y., Chang F., Chen J., Sun X., Cheng D., Huang X., Wu F., Zhang Q., Cai P., Yin P., Zhang L., Tang P. (2024). Histone deacetylase inhibition enhances extracellular vesicles from muscle to promote osteogenesis via miR-873-3p. Signal Transduct. Targeted Ther..

[42] Yan J., Herzog J.W., Tsang K., Brennan C.A., Bower M.A., Garrett W.S., Sartor B.R., Aliprantis A.O., Charles J.F. (2016). Gut microbiota induce IGF-1 and promote bone formation and growth. Proc. Natl. Acad. Sci. U. S. A..

[43] Li Q., Wang Y., Zhang G., Su R., Qi W. (2023). Biomimetic mineralization based on self-assembling peptides. Chem. Soc. Rev..

[44] Kadapure S.A., Deshannavar U.B., Katageri B.G., Kadapure P.S. (2023). A systematic review on MICP technique for developing sustainability in concrete. Eur. J. Environ. Civil Eng..

[45] Gomes A.L.S., Becker-Kerber B., Osés G.L., Prado G., Becker Kerber P., de Barros G.E.B., Galante D., Rangel E., Bidola P., Herzen J., Pfeiffer F., Rizzutto M.A., Pacheco M.L.A.F. (2019). Paleometry as a key tool to deal with paleobiological and astrobiological issues: some contributions and reflections on the Brazilian fossil record. Int. J. Astrobiol..

[46] Xie Y., Xu X., Tang R. (2010). Influence of viscosity on the phase transformation of amorphous calcium carbonate in fluids: an understanding of the medium effect in biomimetic mineralization. Sci. China Chem..

[47] Peled-Kamar M., Hamilton P., Wilt F.H. (2002). Spicule matrix protein LSM34 is essential for biomineralization of the sea urchin spicule. Exp. Cell Res..

[48] Ramadoss R., Padmanaban R., Subramanian B. (2022). Role of bioglass in enamel remineralization: existing strategies and future prospects-A narrative review. J. Biomed. Mater. Res. B Appl. Biomater..

[49] Hashimoto M., Takadama H., Mizuno M., Kokubo T. (2007). Mechanical properties and apatite forming ability of TiO2 nanoparticles/high density polyethylene composite: effect of filler content. J. Mater. Sci. Mater. Med..

[50] Hu L., Zheng H., Wu L., Zhang Z., Yu Q., Tian Y., He G. (2023). Experimental study on the effect of an organic matrix on improving the strength of tailings strengthened by MICP. Materials.

[51] Gower L.B. (2008). Biomimetic model systems for investigating the amorphous precursor pathway and its role in biomineralization. Chem. Rev..

[52] Schuitemaker A., Aufort J., Koziara K.B., Demichelis R., Raiteri P., Gale J.D. (2021). Simulating the binding of key organic functional groups to aqueous calcium carbonate species. Phys. Chem. Chem. Phys..

[53] Takada A., Kadokawa J. (2015). Fabrication and characterization of polysaccharide ion gels with ionic liquids and their further conversion into value-added sustainable materials. Biomolecules.

[54] Tang S., Dong Z., Ke X., Luo J., Li J. (2021). Advances in biomineralization-inspired materials for hard tissue repair. Int. J. Oral Sci..

[55] Bayer I.S. (2023). Controlled drug release from nanoengineered polysaccharides. Pharmaceutics.

[56] Chen S.K., Wang X., Guo Y.Q., Song X.X., Yin J.Y., Nie S.P. (2023). Exploring the partial degradation of polysaccharides: structure, mechanism, bioactivities, and perspectives. Compr. Rev. Food Sci. Food Saf..

[57] Wang Q.C., Zhao X., Pu J.H., Luan X.H. (2016). Influences of acidic reaction and hydrolytic conditions on monosaccharide composition analysis of acidic, neutral and basic polysaccharides. Carbohydr. Polym..

[58] Wang Z., Zheng Y., Lai Z., Hu X., Wang L., Wang X., Li Z., Gao M., Yang Y., Wang Q., Li N. (2024). Effect of monosaccharide composition and proportion on the bioactivity of polysaccharides: a review. Int. J. Biol. Macromol..

[59] Badel S., Bernardi T., Michaud P. (2011). New perspectives for lactobacilli exopolysaccharides. Biotechnol. Adv..

[60] Salazar N., Gueimonde M., de Los Reyes-Gavilán C.G., Ruas-Madiedo P. (2016). Exopolysaccharides produced by lactic acid bacteria and bifidobacteria as fermentable substrates by the intestinal microbiota. Crit. Rev. Food Sci. Nutr..

[61] Schaeffer D.J., Krylov V.S. (2000). Anti-HIV activity of extracts and compounds from algae and Cyanobacteria. Ecotoxicol. Environ. Saf..

[62] Posocco B., Dreussi E., De Santa J., Toffoli G., Abrami M., Musiani F., Grassi M., Farra R., Tonon F., Grassi G., Dapas B. (2015). Polysaccharides for the delivery of Antitumor drugs. Materials.

[63] Pi Y., Fang M., Li Y., Cai L., Han R., Sun W., Jiang X., Chen L., Du J., Zhu Z., Li X. (2024). Interactions between Gut microbiota and natural bioactive polysaccharides in metabolic diseases: review. Nutrients.

[64] Niu Y., Liu W., Fan X., Wen D., Wu D., Wang H., Liu Z., Li B. (2023). Beyond cellulose: pharmaceutical potential for bioactive plant polysaccharides in treating disease and gut dysbiosis. Front. Microbiol..

[65] Gong P., Long H., Guo Y., Wang S., Chen F., Chen X. (2022). Isolation, structural characterization, and hypoglycemic activities in vitro of polysaccharides from Pleurotus eryngii. Molecules.

[66] Su L., Feng Y., Wei K., Xu X., Liu R., Chen G. (2021). Carbohydrate-based macromolecular biomaterials. Chem. Rev..

[67] Murphy E.J., Fehrenbach G.W., Abidin I.Z., Buckley C., Montgomery T., Pogue R., Murray P., Major I., Rezoagli E. (2023). Polysaccharides-Naturally occurring immune modulators. Polymers.

[68] Martins Shimojo A.A., Santos Duarte A.D.S., Santos Duarte Lana J.F., Malheiros Luzo Â C., Fernandes A.R., Sanchez-Lopez E., Barbosa Souto E., Andrade Santana M.H. (2019). Association of platelet-rich plasma and auto-crosslinked hyaluronic acid microparticles: approach for orthopedic application. Polymers.

[69] Yang J., Song Y., Yu Y., Yang X., Zhang X., Zhang W. (2024). Research progress on extraction techniques, structure-activity relationship, and biological functional mechanism of berry polysaccharides: a review. Int. J. Biol. Macromol..

[70] Xue H., Hao Z., Gao Y., Cai X., Tang J., Liao X., Tan J. (2023). Research progress on the hypoglycemic activity and mechanisms of natural polysaccharides. Int. J. Biol. Macromol..

[71] Liu M., Cai M., Ding P. (2021). Oligosaccharides from traditional Chinese herbal medicines: a review of chemical diversity and biological activities. Am. J. Chin. Med..

[72] Zhang S., An L., Li Z., Wang H., Shi L., Zhang J., Li Y., Jin D.Q., Tuerhong M., Ohizumi Y., Shuai L., Xu J., Guo Y. (2020). An active heteropolysaccharide from the rinds of Garcinia mangostana Linn.: structural characterization and immunomodulation activity evaluation. Carbohydr. Polym..

[73] Chen Y., Li X.H., Zhou L.Y., Li W., Liu L., Wang D.D., Zhang W.N., Hussain S., Tian X.H., Lu Y.M. (2017). Structural elucidation of three antioxidative polysaccharides from Tricholoma lobayense. Carbohydr. Polym..

[74] Miao M., Yu W.Q., Li Y., Sun Y.L., Guo S.D. (2022). Structural elucidation and activities of cordyceps militaris-Derived polysaccharides: a review. Front. Nutr..

[75] Sheng Z., Liu J., Yang B. (2021). Structure differences of water soluble polysaccharides in Astragalus membranaceus induced by origin and their bioactivity. Foods.

[76] Lu Y., Jia Y., Xue Z., Li N., Liu J., Chen H. (2021). Recent developments in Inonotus obliquus (Chaga mushroom) polysaccharides: isolation, structural characteristics, biological activities and application. Polymers.

[77] Nishiyama Y., Sugiyama J., Chanzy H., Langan P. (2003). Crystal structure and hydrogen bonding system in cellulose i(alpha) from synchrotron X-ray and neutron fiber diffraction. J. Am. Chem. Soc..

[78] Le C.A., Choisnard L., Wouessidjewe D., Putaux J.L. (2018). Polymorphism of crystalline complexes of V-amylose with fatty acids. Int. J. Biol. Macromol..

[79] Katzenellenbogen E., Kocharova N.A., Zatonsky G.V., Shashkov A.S., Korzeniowska-Kowal A., Gamian A., Bogulska M., Knirel Y.A. (2005). Structure of the O-polysaccharide of Hafnia alvei strain PCM 1189 that has hexa- to octasaccharide repeating units owing to incomplete glucosylation. Carbohydr. Res..

[80] Kondakova A.N., Bystrova O.V., Shaikhutdinova R.Z., Ivanov S.A., Dentovskaya S.V., Shashkov A.S., Knirel Y.A., Anisimov A.P. (2009). Structure of the O-polysaccharide of Yersinia pseudotuberculosis O:2b. Carbohydr. Res..

[81] Yu Y., Tyrikos-Ergas T., Zhu Y., Fittolani G., Bordoni V., Singhal A., Fair R.J., Grafmüller A., Seeberger P.H., Delbianco M. (2019). Systematic hydrogen-bond manipulations to establish polysaccharide structure-property correlations. Angew Chem. Int. Ed. Engl..

[82] Parkhomchuk A.A., Kocharova N.A., Białczak-Kokot M., Shashkov A.S., Chizhov A.O., Knirel Y.A., Rozalski A. (2010). Structure of the O-polysaccharide from the lipopolysaccharide of Providencia alcalifaciens O12. Carbohydr. Res..

[83] Pancerz M., Kruk J., Łukasiewicz M., Witek M., Kucharek M., Jaschik J., Ptaszek A. (2021). Red currant pectin: the physicochemical characteristic of pectin solutions in dilute and semi dilute regimes. Food Hydrocoll..

[84] Aquino R.P., Auriemma G., Mencherini T., Russo P., Porta A., Adami R., Liparoti S., Della Porta G., Reverchon E., Del Gaudio P. (2013). Design and production of gentamicin/dextrans microparticles by supercritical assisted atomisation for the treatment of wound bacterial infections. Int. J. Pharm..

[85] Olsson C., Idström A., Nordstierna L., Westman G. (2014). Influence of water on swelling and dissolution of cellulose in 1-ethyl-3-methylimidazolium acetate. Carbohydr. Polym..

[86] Zhu X., Chen J., Wang H., Tu Z., Yin J., Nie S. (2022). Mechanism of viscosity reduction of okra pectic polysaccharide by ascorbic acid. Carbohydr. Polym..

[87] Leroy A., Very E., Birmes P., Yger P., Szaffarczyk S., Lopes R., Outteryck O., Faure C., Duhem S., Grandgenèvre P., Warembourg F., Vaiva G., Jardri R. (2022). Intrusive experiences in posttraumatic stress disorder: treatment response induces changes in the directed functional connectivity of the anterior insula. Neuroimage, Clin..

[88] Caccamo M.T., Zammuto V., Gugliandolo C., Madeleine-Perdrillat C., Spanò A., Magazù S. (2018). Thermal restraint of a bacterial exopolysaccharide of shallow vent origin. Int. J. Biol. Macromol..

[89] Zhu X.M., Xu R., Wang H., Chen J.Y., Tu Z.C. (2020). Structural Properties, Bioactivities, and Applications of Polysaccharides from Okra [Abelmoschus esculentus (L.) Moench]: A Review. J. Agric. Food Chem..

[90] Ullah S., Khalil A.A., Shaukat F., Song Y. (2019). Sources, extraction and biomedical properties of polysaccharides. Foods.

[91] Guo R., Chen M., Ding Y., Yang P., Wang M., Zhang H., He Y., Ma H. (2022). Polysaccharides as potential anti-tumor biomacromolecules -A review. Front. Nutr..

[92] Gao X., Qi J., Ho C.T., Li B., Xie Y., Chen S., Hu H., Chen Z., Wu Q. (2021). Purification, physicochemical properties, and antioxidant activities of two low-molecular-weight polysaccharides from Ganoderma leucocontextum fruiting bodies. Antioxidants.

[93] Liu Z., Sun X. (2020). A critical review of the abilities, determinants, and possible molecular mechanisms of seaweed polysaccharides antioxidants. Int. J. Mol. Sci..

[94] Flórez-Fernández N., Vaamonde-García C., Torres M.D., Buján M., Muíños A., Muiños A., Lamas-Vázquez M.J., Meijide-Faílde R., Blanco F.J., Domínguez H. (2023). Relevance of the extraction stage on the anti-inflammatory action of fucoidans. Pharmaceutics.

[95] Ye J., Chen D., Ye Z., Huang Y., Zhang N., Lui E.M.K., Xue C., Xiao M. (2020). Fucoidan isolated from Saccharina japonica inhibits LPS-Induced inflammation in macrophages via blocking NF-κB, MAPK and JAK-STAT pathways. Mar. Drugs.

[96] Porter G.C., Schwass D.R., Tompkins G.R., Bobbala S.K.R., Medlicott N.J., Meledandri C.J. (2021). AgNP/Alginate Nanocomposite hydrogel for antimicrobial and antibiofilm applications. Carbohydr. Polym..

[97] Pina S., Oliveira J.M., Reis R.L. (2015). Natural-based nanocomposites for bone tissue engineering and regenerative medicine: a review. Adv. Mater..

[98] Otterlei M., Ostgaard K., Skjåk-Braek G., Smidsrød O., Soon-Shiong P., Espevik T. (1991). Induction of cytokine production from human monocytes stimulated with alginate. J. Immunother..

[99] Becker T.A., Kipke D.R. (2002). Flow properties of liquid calcium alginate polymer injected through medical microcatheters for endovascular embolization. J. Biomed. Mater. Res..

[100] Mrudulakumari Vasudevan U., Lee O.K., Lee E.Y. (2021). Alginate derived functional oligosaccharides: recent developments, barriers, and future outlooks. Carbohydr. Polym..

[101] Huang X., Yu W., Gu W., Liang S., Zhou L., Zhang L. (2025). Mimicking natural biomineralization enabling biodegradable and highly lipophobic alginate hydrogels. Carbohydr. Polym..

[102] LogithKumar R., KeshavNarayan A., Dhivya S., Chawla A., Saravanan S., Selvamurugan N. (2016). A review of chitosan and its derivatives in bone tissue engineering. Carbohydr. Polym..

[103] Wang Z., Su J., Ali A., Yang W., Zhang R., Li Y., Zhang L., Li J. (2022). Chitosan and carboxymethyl chitosan mimic biomineralization and promote microbially induced calcium precipitation. Carbohydr. Polym..

[104] Sohrabi M., Khorasani A.S. (2021). Chitosan-based bionanocomposites in bone tissue engineering. Bionanocomposites in Tissue Engineering and Regenerative Medicine.

[105] Feng Y., Cölfen H., Xiong R. (2023). Organized mineralized cellulose nanostructures for biomedical applications. J. Mater. Chem. B.

[106] Klemm D., Heublein B., Fink H.P., Bohn A. (2005). Cellulose: fascinating biopolymer and sustainable raw material. Angew Chem. Int. Ed. Engl..

[107] Becker L.C., Bergfeld W.F., Belsito D.V., Klaassen C.D., Marks J.G., Shank R.C., Slaga T.J., Snyder P.W., Andersen F.A. (2009). Final report of the safety assessment of hyaluronic acid, potassium hyaluronate, and sodium hyaluronate. Int. J. Toxicol..

[108] Fraser J.R., Laurent T.C., Laurent U.B. (1997). Hyaluronan: its nature, distribution, functions and turnover. J. Intern. Med..

[109] He H., Shao C., Mu Z., Mao C., Sun J., Chen C., Tang R., Gu X. (2020). Promotion effect of immobilized chondroitin sulfate on intrafibrillar mineralization of collagen. Carbohydr. Polym..

[110] Hao J.X., Wan Q.Q., Mu Z., Gu J.T., Yu W.W., Qin W., Li Y.T., Wang C.Y., Ma Y.X., Jiao K., Tay F., Niu L. (2023). A seminal perspective on the role of chondroitin sulfate in biomineralization. Carbohydr. Polym..

[111] Wojtas M., Lausch A.J., Sone E.D. (2020). Glycosaminoglycans accelerate biomimetic collagen mineralization in a tissue-based in vitro model. Proc. Natl. Acad. Sci. U. S. A..

[112] Nogueira L.F.B., Cruz M.A.E., de Melo M.T., Maniglia B.C., Caroleo F., Paolesse R., Lopes H.B., Beloti M.M., Ciancaglini P., Ramos A.P., Bottini M. (2023). Collagen/κ-Carrageenan-Based scaffolds as biomimetic constructs for in vitro bone mineralization studies. Biomacromolecules.

[113] Liu S., Han F., Chen P., Zhang R., Tao Y. (2025). Injectable and drug-loaded gelatin methacrylate and carboxymethylated-sulfated xanthan gum hydrogels as biomimetic mineralization constructs. Carbohydr. Polym..

[114] Esposito F., Laezza A., Gargiulo V., Traboni S., Iadonisi A., La Gatta A., Schiraldi C., Bedini E. (2023). Multi-step strategies toward regioselectively sulfated M-Rich alginates. Biomacromolecules.

[115] Sand K.K., Pedersen C.S., Matthiesen J., Dobberschutz S., Stipp S.L.S. (2017). Controlling biomineralisation with cations. Nanoscale.

[116] Hu J.X., Ran J.B., Chen S., Jiang P., Shen X.Y., Tong H. (2016). Carboxylated agarose (CA)-Silk fibroin (SF) dual confluent matrices containing oriented hydroxyapatite (HA) crystals: Biomimetic Organic/Inorganic composites for Tibia repair. Biomacromolecules.

[117] Yang X., Li X., Wu Z., Cao L. (2023). Photocrosslinked methacrylated natural macromolecular hydrogels for tissue engineering: a review. Int. J. Biol. Macromol..

[118] Vieira S., da Silva Morais A., Garet E., Silva-Correia J., Reis R.L., Gonzalez-Fernandez A., Miguel Oliveira J. (2019). Self-mineralizing Ca-enriched methacrylated gellan gum beads for bone tissue engineering. Acta Biomater..

[119] Ilhan G.T., Irmak G., Gumusderelioglu M. (2020). Microwave assisted methacrylation of Kappa carrageenan: a bioink for cartilage tissue engineering. Int. J. Biol. Macromol..

[120] Zhong X., Zhang S., Wang H., Wang M., Feng Z., Su W., Wang J., Liu Z., Ye L. (2024). Dynamic RGD ligands derived from highly mobile cyclodextrins regulate spreading and proliferation of endothelial cells to promote vasculogenesis. Int. J. Biol. Macromol..

[121] Wang Z., Dong L., Han L., Wang K., Lu X., Fang L., Qu S., Chan C.W. (2016). Self-assembled biodegradable nanoparticles and polysaccharides as biomimetic ECM nanostructures for the synergistic effect of RGD and BMP-2 on bone Formation. Sci. Rep..

[122] Huang Y., Luo Q., Li X., Zhang F., Zhao S. (2012). Fabrication and in vitro evaluation of the collagen/hyaluronic acid PEM coating crosslinked with functionalized RGD peptide on titanium. Acta Biomater..

[123] Brun P., Zamuner A., Battocchio C., Cassari L., Todesco M., Graziani V., Iucci G., Marsotto M., Tortora L., Secchi V., Dettin M. (2021). Bio-Functionalized chitosan for bone tissue engineering. Int. J. Mol. Sci..

[124] Zhang Q., Liu Y., Li J., Wang J., Liu C. (2023). Recapitulation of growth factor-enriched microenvironment via BMP receptor activating hydrogel. Bioact. Mater..

[125] Jeon O., Powell C., Solorio L.D., Krebs M.D., Alsberg E. (2011). Affinity-based growth factor delivery using biodegradable, photocrosslinked heparin-alginate hydrogels. J. Contr. Release.

[126] Ye Q., Zhang Y., Dai K., Chen X., Read H.M., Zeng L., Hang F. (2020). Three dimensional printed bioglass/gelatin/alginate composite scaffolds with promoted mechanical strength, biomineralization, cell responses and osteogenesis. J. Mater. Sci. Mater. Med..

[127] Hu J., Wang Z., Miszuk J.M., Zhu M., Lansakara T.I., Tivanski A.V., Banas J.A., Sun H. (2021). Vanillin-bioglass cross-linked 3D porous chitosan scaffolds with strong osteopromotive and antibacterial abilities for bone tissue engineering. Carbohydr. Polym..

[128] El-Kady A.M., Mahmoud E.M., Sayed M., Kamel S.M., Naga S.M. (2023). In-vitro and in-vivo evaluation for the bio-natural Alginate/nano-Hydroxyapatite (Alg/n-HA) injectable hydrogel for critical size bone substitution. Int. J. Biol. Macromol..

[129] Wang Y., Zhou X., Jiang J., Zhao T., Dang J., Hu R., Shen C., Fan Q., Sun D., Zhang M. (2025). Carboxymethyl chitosan-enhanced multi-level microstructured composite hydrogel scaffolds for bone defect repair. Carbohydr. Polym..

[130] Singh B.N., Veeresh V., Mallick S.P., Jain Y., Sinha S., Rastogi A., Srivastava P. (2019). Design and evaluation of chitosan/chondroitin sulfate/nano-bioglass based composite scaffold for bone tissue engineering. Int. J. Biol. Macromol..

[131] Li Y., Li X., Zhu L., Liu T., Huang L. (2025). Chitosan-based biomaterials for bone tissue engineering. Int. J. Biol. Macromol..

[132] Salehi S., Ghomi H., Hassanzadeh-Tabrizi S.A., Koupaei N., Khodaei M. (2025). Antibacterial and osteogenic properties of chitosan-polyethylene glycol nanofibre-coated 3D printed scaffold with vancomycin and insulin-like growth factor-1 release for bone repair. Int. J. Biol. Macromol..

[133] Zhong C., Chu C.C. (2012). Biomimetic mineralization of acid polysaccharide-based hydrogels: towards porous 3-dimensional bone-like biocomposites. J. Mater. Chem..

[134] Nasiripour S., Pishbin F., Seyyed Ebrahimi S.A. (2025). 3D printing of a Self-Healing, bioactive, and dual-cross-linked polysaccharide-based composite hydrogel as a scaffold for bone tissue engineering. ACS Appl. Bio Mater..

[135] Pan P., Yue Q., Li J., Gao M., Yang X., Ren Y., Cheng X., Cui P., Deng Y. (2021). Smart cargo delivery System based on mesoporous nanoparticles for bone disease diagnosis and treatment. Adv. Sci. (Weinh.).

[136] Luo B., Xuan S., Wang X., Ding K., Jin P., Zheng Y., Wu Z. (2025). Liposome/chitosan coating film bioplastic packaging for Litchi fruit preservation. Food Chem..

[137] Li Q., Li C., Yan J., Zhang C., Jiang Y., Hu X., Han L., Li L., Wang P., Zhao L., Zhao Y. (2025). Evenly distributed microporous structure and E7 peptide functionalization synergistically accelerate osteogenesis and angiogenesis in engineered periosteum. Adv. Sci. (Weinh.).

[138] Xu G., Xi L., Huang X., Xie Q., Zhao J., Jiang X., Lu Z., Zheng L. (2025). Anti-aging chitosan/gelatin film crosslinked byα-arbutin for bone regeneration by free radical scavenging to prevent osteoblast senescence. Biomed. Mater..

[139] Doymus B., Pekozer G.G., Onder S. (2025). Enhancing bioactivity of titanium-based materials through Chitosan based coating and calcitriol functionalization. Ann. Biomed. Eng..

[140] Huang L., Wang W., Xian Y., Liu L., Fan J., Liu H., Zheng Z., Wu D. (2023). Rapidly in situ forming an injectable Chitosan/PEG hydrogel for intervertebral disc repair. Mater. Today Bio.

[141] Cao Z., Bai X., Wang C., Ren L., Ma D. (2021). A simple polysaccharide based injectable hydrogel compositing nano-hydroxyapatite for bone tissue engineering. Mater. Lett..

[142] Suresh N., Shanmugavadivu A., Selvamurugan N. (2025). Chitosan-exosome synergy: advanced cell-free scaffold approaches for bone tissue engineering. Int. J. Biol. Macromol..

[143] Kang Y., Xu J., Meng L., Su Y., Fang H., Liu J., Cheng Y.Y., Jiang D., Nie Y., Song K. (2023). 3D bioprinting of dECM/Gel/QCS/nHAp hybrid scaffolds laden with mesenchymal stem cell-derived exosomes to improve angiogenesis and osteogenesis. Biofabrication.

[144] Man K., Brunet M.Y., Federici A.S., Hoey D.A., Cox S.C. (2022). An ECM-Mimetic hydrogel to promote the therapeutic efficacy of osteoblast-derived extracellular vesicles for bone regeneration. Front. Bioeng. Biotechnol..

[145] Nga N.K., Thanh Tam L.T., Ha N.T., Hung Viet P., Huy T.Q. (2020). Enhanced biomineralization and protein adsorption capacity of 3D chitosan/hydroxyapatite biomimetic scaffolds applied for bone-tissue engineering. RSC Adv..

[146] Wu Y., Li H., Li Y. (2021). Biomineralization induced by cells of Sporosarcina pasteurii: mechanisms, applications and challenges. Microorganisms.

[147] Thakur S., H P S A.K., Bairwan R., Yahya E., Jha K., Adnan A., Khan M. (2024). Potential application of polysaccharide-based Aerogel Scaffolds for bone tissue engineering. Adv. Mater. Lett..

[148] Lekhavadhani S., Babu S., Shanmugavadivu A., Selvamurugan N. (2025). Recent progress in alginate-based nanocomposites for bone tissue engineering applications. Colloids Surf. B Biointerfaces.

[149] Diaz-Rodriguez P., Garcia-Trinanes P., Echezarreta Lopez M.M., Santovena A., Landin M. (2018). Mineralized alginate hydrogels using marine carbonates for bone tissue engineering applications. Carbohydr. Polym..

[150] Tambutté S., Holcomb M., Ferrier-Pagès C., Reynaud S., Tambutté É., Zoccola D., Allemand D. (2011). Coral biomineralization: from the gene to the environment. J. Exp. Mar. Biol. Ecol..

[151] Sivakumar P.M., Yetisgin A.A., Demir E., Sahin S.B., Cetinel S. (2023). Polysaccharide-bioceramic composites for bone tissue engineering: a review. Int. J. Biol. Macromol..

[152] Shi W., Li Z., Peng L., Wang X., Zheng F., Su T., Huang Q., Cao L., Zheng A. (2024). Organic-inorganic nHA-Gelatin/Alginate high strength macroporous cryogel promotes bone regeneration. Smart Mater. Med..

[153] Kaith A., Jain N., Kaul S., Nagaich U. (2024). Polysaccharide-infused bio-fabrication: advancements in 3D bioprinting for tissue engineering and bone regeneration. Mater. Today Commun..

[154] Wang J., Wu Y., Li G., Zhou F., Wu X., Wang M., Liu X., Tang H., Bai L., Geng Z., Song P., Shi Z., Ren X., Su J. (2024). Engineering large-scale self-mineralizing bone organoids with bone matrix-inspired hydroxyapatite hybrid bioinks. Adv. Mater..

[155] Salem N.A., ElShebiney S.A., Mabrouk M., Kishta M.S., Galal A.F., Osama L., Beherei H.H. (2025). Enhanced bone regeneration using mesenchymal stem cell-loaded 3D-printed alginate-calcium Titanate scaffolds: a calvarial defect model study. Int. J. Biol. Macromol..

[156] Ghorbani M., Vasheghani-Farahani E., Azarpira N., Hashemi-Najafabadi S., Ghasemi A. (2023). Dual-crosslinked in-situ forming alginate/silk fibroin hydrogel with potential for bone tissue engineering. Biomater. Adv..

[157] Abdian N., Etminanfar M., Hamishehkar H., Sheykholeslami S.O.R. (2024). Incorporating mesoporous SiO(2)-HA particles into chitosan/hydroxyapatite scaffolds: a comprehensive evaluation of bioactivity and biocompatibility. Int. J. Biol. Macromol..

[158] Hussin M.S.F., Mohd Serah A., Azlan K.A., Abdullah H.Z., Idris M.I., Ghazali I., Mohd Shariff A.H., Huda N., Zakaria A.A. (2021). A bibliometric analysis of the global trend of using alginate, gelatine, and hydroxyapatite for bone tissue regeneration applications. Polymers.

[159] Lv S., Lian X., Feng H., Yang B., Liu Z., Fu T., Zhao L., Huang D. (2025). Three-step crosslinking dependent self-bending transformation of a nano-spherical mineralized collagen laden 4D printed sodium alginate scaffold for bone regeneration. Carbohydr. Polym..

[160] Ding W., Ge Y., Zhang T., Zhang C., Yin X. (2024). Advanced construction strategies to obtain nanocomposite hydrogels for bone repair and regeneration. NPG Asia Mater..

[161] Koons G.L., Diba M., Mikos A.G. (2020). Materials design for bone-tissue engineering. Nat. Rev. Mater..

[162] Rahman S.F., Ghiffary M.M., Tampubolon J.Y., Yulianti E.S., Nadhif M.H., Katili P.A., Hanafiah S., Pangesty A.I., Maras M.A.J. (2024). Effect of graphite, graphene oxide, and multi-walled carbon nanotubes on the physicochemical characteristics and biocompatibility of chitosan/hyaluronic acid/hydroxyapatite scaffolds for tissue engineering applications. J. Sci. Adv. Mater. Devices.

[163] Sarita, Dayaram P.M., Rai A.K., Tewari R.P., Dutta P.K. (2024). Synthesis and characterization of injectable chitosan, hyaluronic acid, and hydroxyapatite blend hydrogel aimed at bone tissue engineering application. Bull. Mater. Sci..

[164] Kim J.W., Han Y.S., Lee H.M., Kim J.K., Kim Y.J. (2021). Effect of morphological characteristics and biomineralization of 3D-Printed Gelatin/Hyaluronic Acid/Hydroxyapatite composite scaffolds on bone tissue regeneration. Int. J. Mol. Sci..

[165] Rajan Unnithan A., Ramachandra Kurup Sasikala A., Park C.H., Kim C.S. (2017). A unique scaffold for bone tissue engineering: an osteogenic combination of graphene oxide–hyaluronic acid–chitosan with simvastatin. J. Ind. Eng. Chem..

[166] Lavrador P., Moura B.S., Almeida-Pinto J., Gaspar V.M., Mano J.F. (2025). Engineered nascent living human tissues with unit programmability. Nat. Mater..

[167] Öfkeli F., Demir D., Bölgen N. (2021). Biomimetic mineralization of chitosan/gelatin cryogels and in vivo biocompatibility assessments for bone tissue engineering. J. Appl. Polym. Sci..

[168] Subramaniam S., Fang Y.H., Sivasubramanian S., Lin F.H., Lin C.P. (2016). Hydroxyapatite-calcium sulfate-hyaluronic acid composite encapsulated with collagenase as bone substitute for alveolar bone regeneration. Biomaterials.

[169] Hachinohe Y., Taira M., Hoshi M., Yoshida D., Hatakeyama W., Sawada T., Kondo H. (2023). Self-Prepared hyaluronic Acid/Alkaline gelatin composite with nano-hydroxyapatite and bone morphogenetic protein for cranial bone Formation. Int. J. Mol. Sci..

[170] Kim H.D., Lee E.A., An Y.H., Kim S.L., Lee S.S., Yu S.J., Jang H.L., Nam K.T., Im S.G., Hwang N.S. (2017). Chondroitin sulfate-based biomineralizing surface hydrogels for bone tissue engineering. ACS Appl. Mater. Interfaces.

[171] Schneiders W., Reinstorf A., Biewener A., Serra A., Grass R., Kinscher M., Heineck J., Rehberg S., Zwipp H., Rammelt S. (2009). In vivo effects of modification of hydroxyapatite/collagen composites with and without chondroitin sulphate on bone remodeling in the sheep tibia. J. Orthop. Res..

[172] Dinoro J., Maher M., Talebian S., Jafarkhani M., Mehrali M., Orive G., Foroughi J., Lord M.S., Dolatshahi-Pirouz A. (2019). Sulfated polysaccharide-based scaffolds for orthopaedic tissue engineering. Biomaterials.

[173] Goonoo N., Khanbabaee B., Steuber M., Bhaw-Luximon A., Jonas U., Pietsch U., Jhurry D., Schonherr H. (2017). kappa-Carrageenan enhances the biomineralization and osteogenic differentiation of electrospun polyhydroxybutyrate and polyhydroxybutyrate valerate fibers. Biomacromolecules.

[174] Farokhi M., Mottaghitalab F., Samani S., Shokrgozar M.A., Kundu S.C., Reis R.L., Fatahi Y., Kaplan D.L. (2018). Silk fibroin/hydroxyapatite composites for bone tissue engineering. Biotechnol. Adv..

[175] Kikionis S., Ioannou E., Aggelidou E., Tziveleka L.-A., Demiri E., Bakopoulou A., Zinelis S., Kritis A., Roussis V. (2021). The marine polysaccharide Ulvan confers potent osteoinductive capacity to PCL-Based scaffolds for bone tissue engineering applications. Int. J. Mol. Sci..

[176] Hu Y., Chen J., Fan T., Zhang Y., Zhao Y., Shi X., Zhang Q. (2017). Biomimetic mineralized hierarchical hybrid scaffolds based on in situ synthesis of nano-hydroxyapatite/chitosan/chondroitin sulfate/hyaluronic acid for bone tissue engineering. Colloids Surf. B Biointerfaces.

[177] Murugan S.S., Anil S., Venkatesan J. (2022). Polysaccharides-based nanoparticles for bone tissue engineering. Polysaccharide Nanoparticles.

[178] Liu Y., Shi C., Ming P., Yuan L., Jiang X., Jiang M., Cai R., Lan X., Xiao J., Tao G. (2024). Biomimetic fabrication of sr-silk fibroin co-assembly hydroxyapatite based microspheres with angiogenic and osteogenic properties for bone tissue engineering. Mater. Today Bio.

[179] Arif M.D., Hoque M.E., Rahman M.Z., Shafoyat M.U. (2024). Emerging directions in green nanomaterials: synthesis, physicochemical properties and applications. Mater. Today Commun..

[180] Chen Y., Gan W., Cheng Z., Zhang A., Shi P., Zhang Y. (2024). Plant molecules reinforce bone repair: novel insights into phenol-modified bone tissue engineering scaffolds for the treatment of bone defects. Mater. Today Bio.

[181] Maharjan B., Park J., Kaliannagounder V.K., Awasthi G.P., Joshi M.K., Park C.H., Kim C.S. (2021). Regenerated cellulose nanofiber reinforced chitosan hydrogel scaffolds for bone tissue engineering. Carbohydr. Polym..

[182] Nourany M., Makaremy A., Bazrpash S., Hosseini S. (2025). Bimodal macroporous 3D scaffolds based on compatibilized PCL and PLA blend using PCL-PEG-PCL block copolymers and cellulose nanocrystals for osteogenic differentiation of hMSCs. Int. J. Biol. Macromol..

[183] Mousa H.M., Hussein K.H., Sayed M.M., Abd El-Rahman M.K., Woo H.-M. (2021). Development and characterization of Cellulose/Iron acetate nanofibers for bone tissue engineering applications. Polymers.

[184] Canas-Gutierrez A., Toro L., Fornaguera C., Borros S., Osorio M., Castro-Herazo C., Arboleda-Toro D. (2023). Biomineralization in three-dimensional scaffolds based on bacterial nanocellulose for bone tissue engineering: feature characterization and stem cell differentiation. Polymers.

[185] Liu X., Hu H., Ma J., Wang B. (2025). Mineralized cellulose nanofibers reinforced bioactive hydrogel remodels the osteogenic and angiogenic microenvironment for enhancing bone regeneration. Carbohydr. Polym..

[186] Izawa H., Nishino S., Maeda H., Morita K., Ifuku S., Morimoto M., Saimoto H., Kadokawa J. (2014). Mineralization of hydroxyapatite upon a unique xanthan gum hydrogel by an alternate soaking process. Carbohydr. Polym..

[187] Pangon A., Saesoo S., Saengkrit N., Ruktanonchai U., Intasanta V. (2016). Hydroxyapatite-hybridized chitosan/chitin whisker bionanocomposite fibers for bone tissue engineering applications. Carbohydr. Polym..

[188] Sun J., Gao R., Qin N., Yang J. (2025). A BMP-2 sustained-release scaffold accelerated bone regeneration in rats via the BMP-2 consistent activation maintained by a non-sulfate polysaccharide. Biomed. Mater..

[189] Zhu X., Wang C., Bai H., Zhang J., Wang Z., Li Z., Zhao X., Wang J., Liu H. (2023). Functionalization of biomimetic mineralized collagen for bone tissue engineering. Mater. Today Bio.

[190] Zhou L., Zhao Z., Banitaba S.N., Khademolqorani S., Han X., Chen G. (2025). Multipurpose triadic MXene/garlic/gellan gum-based architecture in the horizon of bone tissue regeneration. Nanoscale.

[191] Singh A., Kumar S., Acharya T.K., Goswami C., Goswami L. (2022). Application of nanohydroxyapatite-polysaccharide based biomaterial for bone cell mineralization in tissue engineering. Mater. Today Commun..

[192] Sivakumar P.M., Yetisgin A.A., Sahin S.B., Demir E., Cetinel S. (2022). Bone tissue engineering: anionic polysaccharides as promising scaffolds. Carbohydr. Polym..

[193] Huang Y., Zhao Z., Yang Y., Mao R., Li D., Luo F., Wang K., Fan Y., Zhang X. (2025). Synergistic peptide-organic matrix enhances mineralization of biomimetic scaffolds for bone regeneration. Mater. Horiz..

[194] Xu L., Kang H., Wei W., Goto T., Wu X., Dai H. (2024). Freezing, salting‐out and mineralization — a simple, universal and modular strategy for constructing mineralized hydrogels. Adv. Funct. Mater..

[195] Wang L., Li D., Huang Y., Mao R., Zhang B., Luo F., Gu P., Song P., Ge X., Lu J., Yang X., Fan Y., Zhang X., Wang K. (2023). Bionic mineralized 3D‐Printed scaffolds with enhanced in situ mineralization for cranial bone regeneration. Adv. Funct. Mater..

[196] Han X., Saiding Q., Cai X., Xiao Y., Wang P., Cai Z., Gong X., Gong W., Zhang X., Cui W. (2023). Intelligent vascularized 3D/4D/5D/6D-Printed tissue scaffolds. Nano-Micro Lett..

[197] Chen A., Su J., Li Y., Zhang H., Shi Y., Yan C., Lu J. (2023). 3D/4D printed bio-piezoelectric smart scaffolds for next-generation bone tissue engineering. Int. J. Extrem. Manuf..

[198] Liu C., Xu M., Wang Y., Yin Q., Hu J., Chen H., Sun Z., Liu C., Li X., Zhou W., Liu H. (2024). Exploring the potential of hydroxyapatite-based materials in biomedicine: a comprehensive review. Mater. Sci. Eng. R Rep..

[199] Lu Z., Gao W., Liu F., Cui J., Feng S., Liang C., Guo Y., Wang Z., Mao Z., Zhang B. (2024). Vat photopolymerization based digital light processing 3D printing hydrogels in biomedical fields: key parameters and perspective. Addit. Manuf..

[200] Xuan L., Hou Y., Liang L., Wu J., Fan K., Lian L., Qiu J., Miao Y., Ravanbakhsh H., Xu M., Tang G. (2024). Microgels for cell delivery in tissue engineering and regenerative med icine. Nano-Micro Lett..

